# Interleukin-1 as Innate Mediator of T Cell Immunity

**DOI:** 10.3389/fimmu.2020.621931

**Published:** 2021-01-27

**Authors:** Bram Van Den Eeckhout, Jan Tavernier, Sarah Gerlo

**Affiliations:** ^1^ VIB-UGent Center for Medical Biotechnology, VIB, Ghent, Belgium; ^2^ Department of Biomolecular Medicine, Ghent University, Ghent, Belgium; ^3^ Orionis Biosciences BV, Ghent, Belgium

**Keywords:** interleukin-1, dendritic cells, CD4^+^ T cells, CD8^+^ T cells, cellular adjuvant, vaccination, cancer immunotherapy

## Abstract

The three-signal paradigm tries to capture how the innate immune system instructs adaptive immune responses in three well-defined actions: (1) presentation of antigenic peptides in the context of MHC molecules, which allows for a specific T cell response; (2) T cell co-stimulation, which breaks T cell tolerance; and (3) secretion of polarizing cytokines in the priming environment, thereby specializing T cell immunity. The three-signal model provides an empirical framework for innate instruction of adaptive immunity, but mainly discusses STAT-dependent cytokines in T cell activation and differentiation, while the multi-faceted roles of type I IFNs and IL-1 cytokine superfamily members are often neglected. IL-1α and IL-1β are pro-inflammatory cytokines, produced following damage to the host (release of DAMPs) or upon innate recognition of PAMPs. IL-1 activity on both DCs and T cells can further shape the adaptive immune response with variable outcomes. IL-1 signaling in DCs promotes their ability to induce T cell activation, but also direct action of IL-1 on both CD4^+^ and CD8^+^ T cells, either alone or in synergy with prototypical polarizing cytokines, influences T cell differentiation under different conditions. The activities of IL-1 form a direct bridge between innate and adaptive immunity and could therefore be clinically translatable in the context of prophylactic and therapeutic strategies to empower the formation of T cell immunity. Understanding the modalities of IL-1 activity during T cell activation thus could hold major implications for rational development of the next generation of vaccine adjuvants.

## Introduction

Innate immune cells represent the first line of defense in response to an injury or a pathogen encounter. Next to this, specialized innate immune cells called antigen-presenting cells (APCs), including dendritic cells (DCs), also collect peripheral information on the source of the antigen and transfer this data to cells of the adaptive immune system. This information transfer happens over three major routes: (1) presentation of antigenic peptides to antigen-specific T cells, resulting in an antigen-specific response; (2) co-stimulation, which can be perceived as qualitative information on the context of the antigen and allows for breaking tolerance; and (3) release of priming cytokines that polarize the T cell response, as such raising a tailor-made reaction to the encountered threat. This so-called “three-signal paradigm” places activation of the adaptive immune system under innate control and is currently a well-established paradigm in immunobiology (1). In order to perform these essential functions, innate immune cells are equipped with germline-encoded extra- and intracellular pathogen-recognition receptors (PRRs). These allow the innate immune system to recognize evolutionary conserved “non-self” microbial components (pathogen-associated molecular patterns (PAMPs), *e.g.* bacterial cell wall components or viral nucleic acids) and host-derived damage-associated molecular patterns (DAMPs), *e.g.* extracellular ATP and monosodium urate crystals) (2, 3).

The T cell reaction that results from this three-leveled instruction can be regarded as “productive” adaptive immunity: a measurable and antigen-specific response that is characterized by activated T cells that undergo clonal expansion and produce effector cytokines, but which is not necessarily “protective” upon pathogen encounter (4). Vaccines aim to mimic natural infections, but progression from productive to actually protective T cell responses remains a major challenge for the vaccine field, where most strategies are still dependent on the generation of long-lasting neutralizing antibodies (4, 5). Nevertheless, there is a dire need for T cell-inducing vaccines, as both CD4^+^ and CD8^+^ T cell responses have been deemed crucial for protection against different pathogens, such as *Plasmodium falciparum*, HIV and *Mycobacterium tuberculosis*, and for successful treatment of cancer. Although a lot of research has already been dedicated to develop vaccines that provide protective T cell immunity, the majority of these efforts were found to only result in low-level and non-protective responses (5, 6).

A limitation of the three-signal model, as it stands today, is its oversimplification of how the innate immune system controls and sculpts T cell responses. Indeed, innate immune cells provide extra clues on the origin of the antigen outside of the three major information routes addressed above. In their 2017 opinion piece (4), Jain and Pasare discuss different limitations of the current three-signal model and argue that its revisitation is needed in order to successfully translate these principles for the generation of protective T cell responses in different contexts. For instance, the fact that pathogens carry ligands for multiple PRRs infers cross-talk between different PRRs in innate immune cells, which can alter the outcome of their activation. Eventual occurrence of co-infection should also be considered, as this was found to possibly influence the T cell reaction mounted in response to the initial infection. Recent work has also demonstrated that the host’s history of prior infection, which is often neglected in mouse models, and the composition of the commensal microbiota and the metabolites derived thereof can sculpt the behavior of innate and adaptive immune cells (4).

Another issue is that during T cell priming, the cytokine environments that are formed are probably more complex and include other players next to the prototypical priming cytokines. The innate cytokines produced during the initiation of T cell immunity can be roughly categorized based on their dependence on STATs or MyD88 for signaling. Cytokines that signal *via* STATs are well-studied as these induce the expression of lineage-specific transcription factors and as such drive differentiation of CD4^+^ T cells towards different phenotypes (**Table 1**). This plasticity is not restricted to CD4^+^ T cells as also naive CD8^+^ T cells can acquire different phenotypes during priming, not all of which with cytotoxic functionality and the capacity to produce IFN-γ. These so-called T_C_ subsets mirror the different CD4^+^ T cell phenotypes and are also shaped by the same environmental STAT-signaling cytokines as for their CD4^+^ counterparts (30). The roles of MyD88-dependent cytokines in the priming environment, including interleukin-1 (IL-1) cytokine superfamily members, are rather neglected as the focus of the three-signal model primarily lies on STAT-signaling cytokines that polarize the T cell response (4). However, the pro-inflammatory activities of the IL-1 cytokines IL-1α and IL-1β take on a central role during activation of the innate immune system and innate instruction of adaptive immunity (1). IL-1α is released in the extracellular environment as a consequence of cellular damage and locally acts as an alarmin. On the other hand, innate immune cells integrate PAMP and DAMP signals and respond by controlled production of IL-1β (31).

**Table 1:** Overview of the described subsets of CD4^+^ T cells discussed in this review.

	CD4^+^ T_H_0	CD4^+^ T_H_1	CD4^+^ T_H_2	CD4^+^ T_H_17	CD4^+^ Treg	CD4^+^ Tfh	CD4^+^ Tfr	CD4^+^ T_H_9
**Polarization**		IL-12 IFN-γ	IL-4	TGF-β IL-6	TGF-β IL-2	IL-6 IL-21		IL-4 TGF-β
**Polarization TF(s)**		STAT4 STAT1	STAT6	STAT3	STAT5	STAT3		IRF4
**Lineage TF(s)**		T-bet	GATA-3	RORγT	FoxP3	Bcl6	FoxP3 Bcl6	
**Lineage commitment**		IL-18	IL-33	IL-1β IL-21 IL-23	RA			
**Effector cytokines**		IFN-γ TNF	IL-4 IL-5 IL-13	IL-17A/F IL-21 IL-22 IL-25 IL-26	IL-10 TGF-β	IL-4 IL-21	IL-10 TGF-β	IL-9
**IL-1R1** IL-1α/β (+) IL-1Ra and IL-38 (-)	low on T_H_0 (7) ↑↑ on T_EM_ (7)	negative(?) (8) positive(?) (9)	negative(?) (8) positive(?) (9)	high (8–11)	negative (12)	high (12)	low (12)	ND
**IL-1R2** Decoy receptor IL-1α/β (-)	ND	ND	ND	ND	positive population of thymic Treg (13) positive (tumor) (14) ↑↑ activation (15)	negative (16)	high (12)	ND
**IL-1R4** IL-33 (+)	low on T_H_0 (17)	negative (resting) (18) low/transient (active) (18)	positive (resting) (18) high (active) (8, 19)	negative (resting) (18)	positive (20, 21)	negative (22)	negative (23)	positive (24)
**IL-1R5** IL-18 (+)	low on T_H_0 (7) ↑↑ on T_EM _(7)	high (8, 25, 26)	negative (27)	ND	positive population of thymic Treg (28)	ND	ND	ND
**IL-1R6** IL-36α/β/γ (+) IL-36Ra and IL-38 (-)	High on T_H_0 (29)	ND	ND	ND	ND	ND	ND	ND

An overview of the differentiation conditions for murine CD4^+^ T cells subsets and their expression of IL-1 receptor superfamily members. This table is limited to signal-competent primary receptors (IL-1R1, IL-1R4, IL-1R5 and IL-1R6) and the decoy receptor IL-1R2. Respective ligands for these receptors chains and whether they elicit signaling (+) or not (−) is indicated. T_EM_ = effector memory T cell (CD44^+^ CD62L^-^ in naive mice and CD45RO^+^CD45RA^−^ in humans); IL-1R4, ST2; IL-1R5, IL-18Rα; ND, no data found with the literature search used in this review; RA, retinoic acid; TF, transcription factor. Note: subsets of CD8^+^ T cells mimic those described for CD4^+^ T cells, but this falls beyond the scope of the current review. We wish to direct interested readers to other review articles (30).

Here, we will review established and new literature in support of a role for IL-1 as innate instructor of adaptive immune responses and elaborate on how IL-1 activity influences three protagonists of the three-signal model: DCs, CD4^+^ and CD8^+^ T cells. It has become clear that IL-1 activity on both the antigen-presenting DC and the T cell, either alone or in synergy with priming cytokines, can contribute to the formation of protective T cell immunity. As such, IL-1α and IL-1β might play a prominent role as innate instructors of adaptive immune responses. Gaining more insight in the environmental context wherein T cell priming takes place may help to advance prophylactic and therapeutic applications (4).

## Interleukin-1

The IL-1 superfamily of ligands and receptors currently comprises 21 members: 11 soluble factors and 10 receptor molecules. Among these soluble factors, seven perform pro-inflammatory actions (IL-1α, IL-1β, IL-18, IL-33, IL-36α, IL-36β, and IL-36γ) and four display anti-inflammatory activities. Two of these anti-inflammatory mediators are receptor agonists (IL-37 and IL-38) and two are receptor antagonists (IL-1Ra and IL-36Ra). The IL-1 receptor superfamily is organized in several (overlapping) subgroups: ligand-binding receptor proteins (IL-1R1, IL-1R2, IL-1R4, IL-1R5, and IL-1R6), accessory chains (IL-1R3 and IL-1R7), molecules that inhibit signaling (IL-1R2, IL-1R8, and IL-18 binding protein) and orphan receptors (IL-1R9 and IL-1R10). In this review, we solely discuss the IL-1 cytokines IL-1α and IL-1β. These cytokines exert their biology *via* the same binary receptor complex that contains the primary receptor IL-1R1 and the accessory chain IL-1R3 (31–33). From here on, this is the IL-1 receptor (IL-1R) complex referred to in this review.

**Figure 1:**
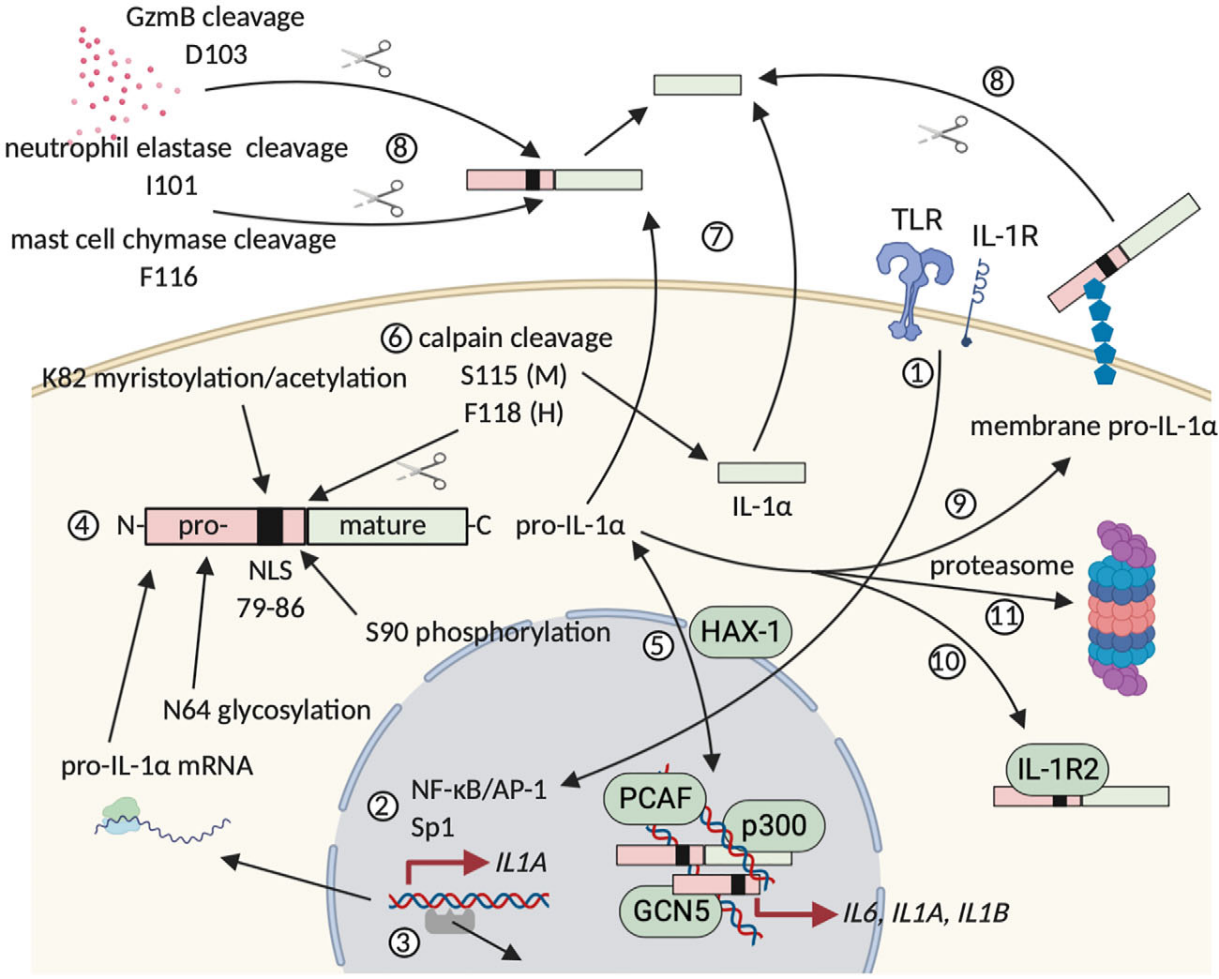
Synthesis, modification and extracellular release of human (pro-)IL-1α. (1) and (2) Inflammatory triggers can induce pro-IL-1α expression, whereas homeostatic expression is regulated by housekeeping transcription factors, such as Sp1. (3) Alternatively, expression can be initiated following DNA release of negative regulators. (4) Pro-IL-1α is modified post-translationally. (5) Pro-IL-1α continuously shuttles between cytosol and nucleus and nuclear translocation is mediated *via* HAX-1, which binds to the pro-IL-1α NLS. Binding of pro-IL-1α to chromatin sequesters the cytokine in the nucleus, whereas interaction with histone modifying enzymes mediates pro-inflammatory gene expression. (6) Calpain cleaves pro-IL-1α in the cytosol, leading to the formation of mature and fully biologically active IL-1α. (7) and (8) Following accidental cell death or damage, (pro-)IL-1α is released extracellularly, where maturation by cleavage is mediated by granzyme B, neutrophil elastases and mast cell chymases. (9) Post-translational modification can allow for extracellular membrane anchorage of pro-IL-1α. (10) A unique intracellularly occurring form of IL-1R2 can neutralize and sequester pro-IL-1α in the cytosol, possibly by masking its NLS. (11) Turnover of (pro-)IL-1α and the cleaved N-terminal pro-piece is mediated in the proteasome. Figure adapted from (35). Created with BioRender.com.

IL-1α is constitutively expressed as a 31 kDa precursor protein (pro-IL-1α) with a basal level of pro-inflammatory activity (31, 34). Expression of pro-IL-1α varies over different cell types, but cells lining body barriers, such as epithelial and endothelial cells, contain relatively high cytoplasmic levels of this protein under homeostatic conditions (35). Alternatively, expression of the IL-1α precursor protein is inducible and potential triggers include pro-inflammatory mediators (*e.g.* IL-1α itself and IL-1β) and growth or stress-associated factors (*e.g.* TLR agonists, such as LPS) (35, 36). Pro-IL-1α comprises an N-terminal precursor part, which is linked to the mature and fully biologically active IL-1α cytokine (31, 34). This N-terminus contains the LKKRRL nuclear localization sequence (NLS) that allows for translocation of pro-IL-1α to the nucleus (37). Nuclear translocation is regulated by interaction with HCLS1-associated protein X (HAX)-1 (38). In the nucleus, pro-IL-1α can directly regulate the expression of pro-inflammatory chemokines and interact with histone modifying enzymes, such as the acetyltransferases p300, p300/CBP-associated factor (PCAF) and GCN5 (39, 40). Pro-IL-1α continuously shuttles between the nucleus and the cytosol within nanoseconds and its commitment to a subcellular compartment is determined by environmental cues. When the cell receives a pro-apoptotic trigger, pro-IL-1α rapidly migrates into the nucleus and binds tightly to the chromatin, while upon necrotic cell death, the precursor is found exclusively in the cytosol (41). As such, accidental cell death results in far more pro-inflammatory lysates and under these conditions, pro-IL-1α performs the role of a classical DAMP, hence its “alarmin” alias (35, 41). Pro-IL-1α can be further processed intra- and extracellularly by different proteases, including calpain and neutrophil elastases. These cleave the N-terminal precursor from the mature IL-1α protein, which augments biological activity (34). Next to a nuclear and an extracellular role, pro-IL-1α can also appear bound on the cell surface following stimulation of hematopoietic and non-hematopoietic cells, including fibroblasts and endothelial cells, with pro-inflammatory mediators (35, 42). Pro-IL-1α probably becomes glycosylated intracellularly and subsequently anchors to the cell membrane *via* a lectin-like interaction. This membrane-associated pro-IL-1α has the ability to interact with IL-1R complexes on neighboring cells and as such mediate paracrine signaling (34, 35). Membrane-bound pro-IL-1α can be shaved from the cell surface by extracellular proteases, as such allowing for its release in the environment (35, 43). **Figure 1** summarizes the expression, post-translational modification and subsequent processing of (pro-)IL-1α.

**Figure 2:**
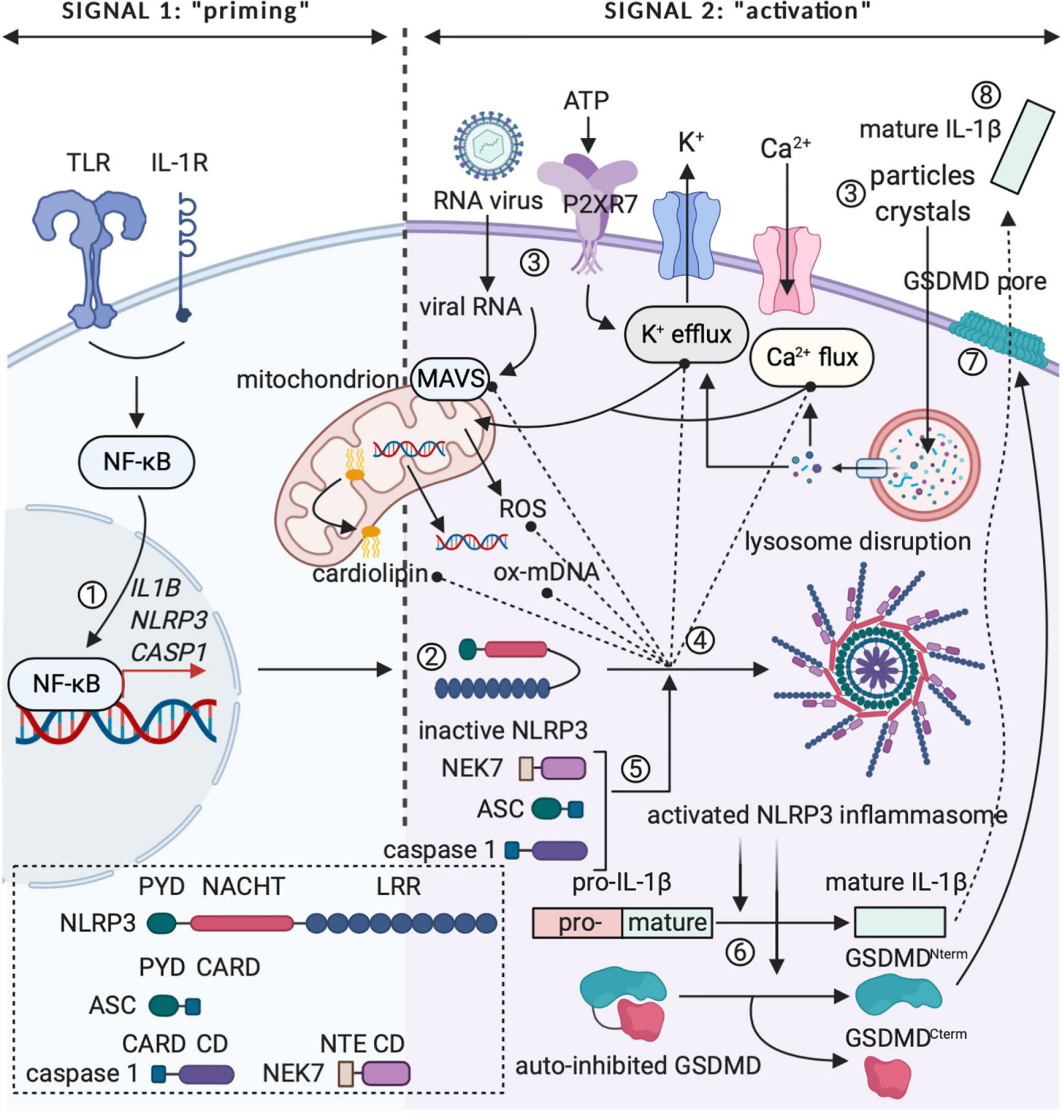
The two-step paradigm of controlled IL-1β release. (1) Signal 1 or “priming” involves expression of pro-IL-1β, NLRP3 and caspase 1 following triggering of pro-inflammatory receptor complexes (*e.g.* TLR4 or IL-1R) and activation of the transcription factor NF-κB. (2) NLRP3’s LRR falls back onto the NACHT domain, rendering the protein inactive in the cytoplasm. (3) Signal 2 or “activation” can be mediated by PAMPs (*e.g.* viral RNA) or DAMPs (*e.g.* extracellular ATP, extracellular particles and crystal complexes). Viral RNA can activate mitochondrial antiviral signaling (MAVS) protein. ATP is detected *via* the P2RX7 receptor and extracellular particles and crystal complexes can induce intracellular lysosome disruption, leading to changes in Ca^2+^ flux and K^+^ efflux. Intracellular changes in ion concentration can influence mitochondria, as such inducing release of ROS and oxidized mtDNA or relocalization of cardiolipin to the outer mitochondrial membrane. (4) and (5) Relocalized cardiolipin, oxidized mDNA, activated MAVS protein and changes in cytosolic ion concentrations can induce conformational changes in NLRP3 and lead to formation of the activated NLRP3 inflammasome after recruitment of NEK7, ASC and caspase 1. (6) Activated caspase 1 cleaves immature pro-IL-1β to mature and fully biologically active IL-1β. In addition, caspase 1 can cleave the GSDMD protein. (7) GSDMD^Nterm^ integrates in the membrane as a pore, which induces pyroptosis and represents a putative way for IL-1β release. CD, catalytic domain; NTE, N-terminal extension. Figure adapted from (50). Created with BioRender.com.

IL-1β is a circulating factor, for which the expression is not constitutive, but tightly regulated. A major source of IL-1β are cells of myeloid origin, which express the cytokine as a cytoplasmic 31 kDa precursor protein (pro-IL-1β) that needs to be converted into a mature form to allow for activity (31, 44, 45). The conventional mechanism leading to activation of pro-IL-1β is proteolytic cleavage in the N-terminal precursor region by caspase 1 (46, 47). Caspase 1 houses in the cytoplasm as an inactive protease that depends on further proteolytic processing to become activated, which takes place in multimolecular protein complexes called inflammasomes (34, 48, 49). Processing of inactive pro-caspase 1 into fully biologically active caspase 1 can be mediated by different canonical inflammasomes, the NLRP3 inflammasome unarguably being the most extensively studied (48–50). Assembly and activation of the NLRP3 inflammasome depends on two distinct signals: (1) an NF-κB-activating stimulus, such as a cytokine or TLR agonist (PAMP), which induces the expression of pro-IL-1β, NLRP3 and caspase 1 in the cytosol (“priming”); and (2) an NLRP3-activating agent that leads to the actual assembly of the functional inflammasome complex (“activation”) (50, 51). This signal two can be rendered by different bacterial, viral and fungal PAMPs (*e.g.* peptidoglycan, single and double stranded RNA and zymosan) and a multitude of DAMPs (*e.g.* extracellular ATP and monosodium urate crystals) (50, 52–56). Alternatively, cleavage of pro-IL-1β can be mediated by caspase 8, a caspase family protease that is commonly associated with pro-apoptotic signaling *via* TNF receptor family members, such as Fas and TRAIL (57). Although unprocessed IL-1β is usually not found in the extracellular space, it can be found outside of the cell as a result of uncontrolled cell death following a stressful event or a strong inflammatory trigger (34). Different cleavage sites for neutrophil proteases and mast cell chymases are present within the N-terminal region of pro-IL-1β. Despite the fact that these proteases can individually yield mature and biologically active IL-1β (58, 59), recent work found that protease mixtures from phorbol 12-myristate 13-acetate (PMA)-activated neutrophils can activate pro-IL-1α, but surprisingly fail to activate pro-IL-1β (60). As IL-1β contains no leader sequence, the matured cytokine is released in the environment *via* an unconventional secretion mechanism that is independent of the endoplasmic reticulum (ER) and Golgi network (61). Multiple theories on how such a “leaderless” protein is released in the environment have been proposed, one of the earlier working models suggesting that IL-1β could be secreted *via* vesicular mechanisms, such as secretory lysosomes, multivesicular bodies and exosomes, or *via* secretory autophagy (61–64). Recent research into the secretion mechanism of IL-1β focuses on pyroptosis, a rapid and inflammatory form of programmed cell death induced following NLRP3 inflammasome activation. The pore-forming protein gasdermin D (GSDMD) was recently identified as the primary mediator of pyroptosis and its activity was found to depend on maturation by caspase 1 cleavage. Pyroptosis ultimately leads to cell death, which allows for the release of mature IL-1β in the process (65–67). However, GSDMD pores can perform an additional non-pyroptotic role that allows for IL-1β release from viable cells with intact, but porous cell membranes (68–70). In **Figure 2**, the controlled expression of (pro-)IL-1β is illustrated graphically.

**Figure 3:**
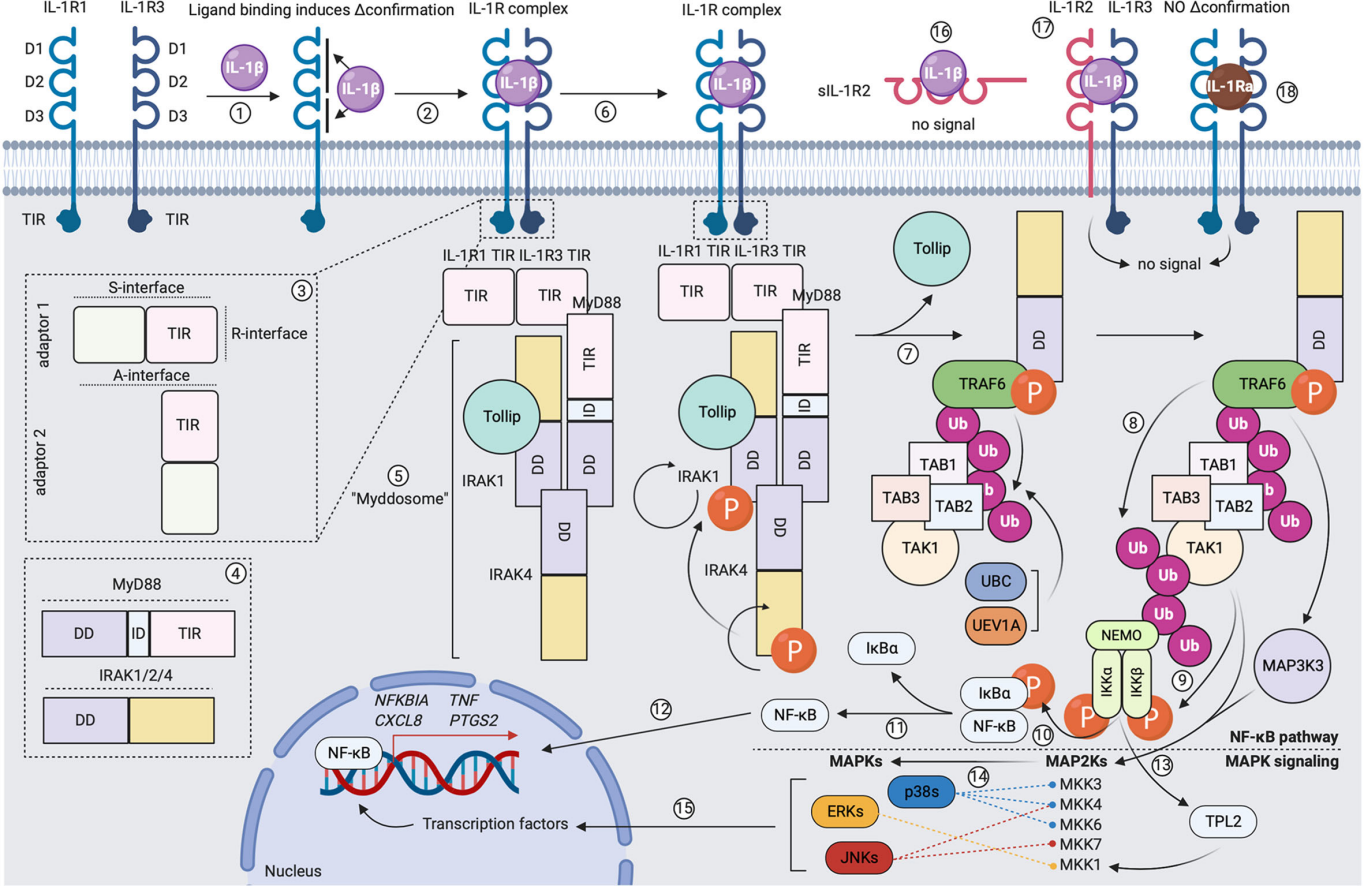
Formation of the signaling-competent IL-1R complex and downstream IL-1 signaling. (1) Extracellular IL-1β makes two contacts with IL-1R1 and induces a conformational change in the primary receptor. (2) This allows IL-1R1 to recruit and bind to co-receptor IL-1R3. (3) Receptor dimerization leads to scaffolding of intracellular receptor TIR domains *via* the R-interface. (4) and (5) Formation of an intracellular TIR scaffold allows for recruitment of the MyD88 adaptor molecule *via* S-interface interactions. MyD88 comprises an N-terminal death domain (DD), an intermediate domain (ID) and a C-terminal TIR domain. IRAK-1, IRAK-4 and/or IRAK-2 are recruited. These molecules comprise an N-terminal DD and a C-terminal catalytic domain. Under steady-state, IRAK-1 is bound to Tollip. As such, the Myddosome is formed. (6) IRAK-4 auto-phosphorylates and gains full activity, in turn activating IRAK-1 by phosphorylation. This induces rapid IRAK-1 auto-phosphorylation. (7) A conformational change in IRAK-1 allows its dissociation from Tollip. Different IRAK-1 oligomers form a scaffold for TRAF6, which becomes activated upon oligomerization. UBC13 and UEV1A facilitate TRAF6 self-poly-ubiquitination (K63-linked). TAB2 and TAB3 recruit TAB1 and the MAP3K TAK1 to the K63-linked poly-ubiquitin chain on TRAF6. (8) TRAF6 poly-ubiquitinates TAK1. (9) Initiation of the canonical NF-κB pathway: NEMO binds to the poly-ubiquitin chain on TAK1 and recruits IKKα and IKKβ. IKKα and IKKβ become activated following phosphorylation by TAK1. (10) and (11) IKKα and IKKβ phosphorylate IκBα, which allows for its dissociation from p50/p65 NF-κB. (12) Free p50/p65 NF-κB translocates to the nucleus and binds to specific κB sites on DNA, as such inducing gene expression. (13) Initiation of the MAPK pathway: the MAP3K MAP3K3 binds the poly-ubiquitin chain on TAK1. MAP3K3 becomes activated following phosphorylation by TRAF6. IKKα and IKKβ activate TPL2 by phosphorylation. MAP3K3, TAK1 and TPL2 activate different MAP2Ks by phosphorylation. (14) MAP2Ks activate the p38, JNK and ERK MAPKs by phosphorylation. (15) In turn, MAPKs activate different transcription factors by phosphorylation, as such inducing gene expression. (16) Binding of IL-1β to sIL-1R2 does not induce signal transduction. (17) Binding of IL-1β to the signaling-incompetent IL-1R complex does not induce signal transduction. (18) Binding of IL-1Ra to the signaling-competent IL-1R complex does not induce signal transduction. Created with BioRender.com.

Receptors within the receptor superfamily generally have a strongly conserved, homologous molecular structure. The extracellular part of these molecules is “question mark”- or “grasping hand”-like shaped and comprises three immunoglobulin (Ig)-like domains (D1, D2 and D3). The transmembrane domain is a single helix that anchors the extracellular portion in the plasma membrane. The intracellular part contains a Toll-IL-1R (TIR) domain, which is responsible for signal transduction (32, 33). Agonists of the IL-1 cytokine superfamily mediate their signaling *via* a three-step mechanism: (1) formation of a binary complex upon binding to a ligand-binding receptor subunit; (2) formation of a ternary complex after inducing a conformational change in the ligand-binding receptor subunit, which allows for recruitment and binding to an accessory chain; (3) juxtaposing the TIR domains of both receptor subunits (if present), which allows for signal transduction (31–33). As mentioned above, IL-1α and IL-1β signal *via* a receptor complex composed of the primary ligand-binding subunit IL-1R1 and the accessory chain IL-1R3. The three extracellular Ig-like domains of IL-1R1 provide two comparably-sized cytokine interaction interfaces. D1 and D2, which are relatively rigid and positioned tightly next to each other, form a first binding interface, while a second contact area is provided by the more flexible D3 (71). The D1/D2 interface has been described to be sufficient for capturing the ligand (72), but the interaction provided by the D3 domain is responsible for the conformational changes in IL-1R1 that allow for recruitment and binding to IL-1R3 (73, 74). The accessory protein binds with its backside to ligand-bound IL-1R1/2, making contacts with D2 and D3 (73, 74). Upon formation of the ternary complex, the intracellular TIR domains of the ligand-binding subunit and the accessory chain juxtapose, as such forming a scaffold structure that allows for recruitment and binding of a sequence of adaptor molecules to the intracellular site of activated receptor complexes, which initiates downstream signal transduction and activation of different transcription factors (32, 33). Downstream IL-1R signaling events are extensively reviewed elsewhere (32, 36) and we summarized the main modalities in **Figure 3**.

A hallmark within the IL-1 superfamily is the tight control over cytokine activity, in particular for IL-1α and IL-1β (see also **Figure 3**). A first level of IL-1 activity regulation is the membrane-bound decoy receptor IL-1R2, which lacks an intracellular TIR domain and is therefore unable to transduce the signal (32, 33). However, cytokine-bound IL-1R2 is still able to recruit and interact with IL-1R3, as such decreasing the availability of this accessory chain on the cell membrane. IL-1R3 sequestering is an important regulatory mechanism, as the availability of surface-expressed IL-1R3 is the rate-limiting step in the formation of IL-1R complexes due to its relatively low abundance compared to the primary receptor chains (75). A second level of regulation is the availability of soluble decoy receptors with the capacity to bind and neutralize circulating cytokines, which can be generated by alternative splicing of the encoding mRNA or cleavage of the receptor protein from the membrane by metalloproteinases (32, 76). Receptor antagonists form a third level of IL-1 activity regulation. IL-1Ra shows a very homologous molecular configuration compared to IL-1α and IL-1β, but carries shorter peptide loops between β-strands 4/5 and 5/6. As such, IL-1Ra cannot bind to D3 of IL-1R1/2 and is therefore unable to induce the conformational change in the receptor subunit that is essential to recruit and interact with the accessory chain (33, 77, 78).

## How IL-1 Affects the Main Players of the Immune Response to Antigen

### IL-1 Signaling in Dendritic Cells Empowers Their Maturation and Facilitates T Cell Priming

Functional maturation of DCs is required for successful priming of naive T cells and initiation of potent antigen-specific adaptive immune responses. Understanding the determinants of efficient DC maturation is therefore critical for the development of clinical applications, which becomes evident from advances made in the field of DC therapy for the treatment of different types of cancer (79). The first generation of such immunotherapies relied on the generation of monocyte-derived DCs (moDCs) from PBMCs after *ex vivo* culture in the presence of GM-CSF and IL-4 (80, 81). These moDCs were subsequently loaded with tumor lysates, tumor-associated antigens (TAAs) or synthetic peptides and administered to patients. However, DC therapy as such only showed a disappointing tumor-regression rate of around 3.3% (82–84). Second-generation therapies expanded on this concept by maturing the moDCs by treatment with LPS, CD40L or cocktails that included TNF, IL-6, IL-1β, PGE_2_ and polyinosinic:polycytidylic acid (poly(I:C)) or combinations thereof. The use of matured DCs further increased the clinical efficacy of these therapies up to 8-15%, depending on the tumor type (79, 85, 86). An alternative approach that bypasses the need for cytokine maturation cocktails involves *ex vivo* electroporation of moDCs with mRNA encoding CD40L, CD70 and constitutively active TLR4, which is referred to as the TriMix formula. Co-delivery of mRNA that encodes for tumor antigens was found to empower T cell responses and trials in melanoma patients revealed that this therapy is safe and establishes durable immunogenic clinical responses (87). Nowadays, the most recent efforts in the field focus on further combinations of these second-generation DC therapies with checkpoint inhibition molecules (*e.g.* anti-CTLA-4 and anti-PD-(L)1), which shows increased overall survival compared with monotherapies in stage III and IV melanoma patients, or chemotherapy (*e.g.* carboplatin and paclitaxel) (79, 88–90).

Maturation of DCs can thus be initiated by triggering PRRs, such as TLR4, or following activation of pro-inflammatory cytokine receptors, including the IL-1R complex. The overlapping effect of LPS and IL-1 on DC maturation results from the fact that both ligands signal *via* TIR domain-containing receptor complexes that share multiple downstream signaling mediators, many of these with a known involvement in the regulation of DC maturation and their capacity to present antigen (91). Signaling downstream of IL-1R activation is graphically represented in **Figure 3**. MyD88^−/−^ mice, for example, fail to mount adaptive immune responses against *Listeria monocytogenes* and are therefore highly susceptible to infection (92, 93). However, restoration of MyD88 signaling in DCs only is sufficient to regain control over the infection (94). Conversely, upon hyperactivation of MyD88 in DCs, mice suffer from severe autoimmune effects, characterized by spontaneous activation of B and T cells, production of autoantibodies and systemic inflammation (95). Also, TRAF6-deficient DCs fail to upregulate CD86 and MHC-II, produce pro-inflammatory cytokines (*e.g.* IL-12 and IL-6) and prime naive T cells following stimulation with CD40L or LPS (96). Further downstream, canonical NF-κB signaling plays a prominent role during maturation of murine and human DCs as NF-κB drives the expression of co-stimulatory molecules and antigen presentation (97–100). Intriguingly, NF-κB signaling in DCs has been suggested to allow for self-tolerance, as steady-state migratory DCs display an enriched alternatively regulated network of NF-κB-instructed genes (101, 102). Among the MAPKs, p38 is described the most extensively in the context of DC activation and maturation. p38 inhibition impairs clustering of DCs with T cells and effector T cell activation (103). Phosphorylation of p38 in DCs correlates with improved uptake and presentation of antigens *via* MHC-I and MHC-II and the upregulation of co-stimulatory molecules (104–106). Studies on the role of JNK and ERK during DC maturation and antigen presentation are more limited. In human DCs, steady-state levels of JNK are notably lower than those of p38 (103). Although p38 and JNK are known to be redundant for many cellular functions, different reports propose that JNK signaling could be negatively regulating presentation of antigens *via* MHC-I and CD1d (107–109). ERK signaling, on the other hand, positively regulates CD1d-mediated presentation of lipid antigens and promotes the survival of matured DCs (91, 97).

**Figure 4:**
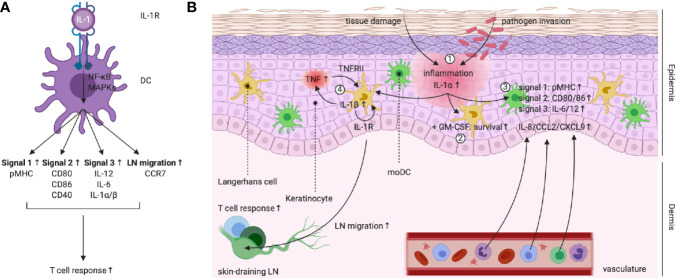
**(A)** Left scheme: IL-1R triggering in DCs empowers their capacity to promote T cell responses. **(B)** IL-1 activity plays a central role during the keratinocyte-LC cross-talk in the skin. (1) Damage to keratinocytes following tissue damage or pathogen invasion leads to the release of (pro-)IL-1α. (2) In synergy with tissue-derived GM-CSF, IL-1R signaling promotes LC survival. (3) IL-1R signaling in moDCs upregulates pMHC (signal 1), expression of co-stimulatory molecules (signal 2), production of priming cytokines (signal 3) and release of chemokines, leading to attraction of neutrophils, monocytes and lymphocytes. (4) PRR and IL-1R signaling promotes LC IL-1β production. IL-1β acts on keratinocytes and induces TNF release, which in turns signals *via* TNFRII on LCs. TNFRII and autocrine IL-1R signaling in LCs enable migration to the skin-draining LNs, where T cell responses can be initiated. Created with BioRender.com.

The capacity of IL-1 to drive DC maturation (**Figure 4A**) was first suggested in 1987 by the group of Ralph Steinman, who demonstrated that DCs pre-treated with low doses of recombinant IL-1 were more potent in stimulating helper T cells compared to unconditioned DCs. In fact, IL-1 was the first cytokine reported to empower the activation of T cells by interfering at the level of the APC (110). Over the decades, different studies have shown that IL-1 signaling is indeed involved during the maturation of different DC subsets by driving the upregulation of several co-stimulatory molecules (*e.g.* CD80, CD86, CD40 and SLAM), release of pro-inflammatory cytokines (*e.g.* IL-12, IL-1α/β and IL-6) and expression of surface MHC (111–114). During steady state, lower numbers of peripheral CD103^+^ DCs are observed in IL-1R1^−/−^ mice compared to wild-type (WT) animals and these cells additionally display a less mature phenotype, indicated by lower surface CD86 expression. Upon infection with influenza A virus (IAV), IL-1R1^−/−^ CD103^+^ DCs in the lung fail to upregulate CCR7 and show an impaired capacity to migrate and accumulate in lung-draining lymph nodes (LNs) (115). Conversely, IL-1Ra^−/−^ DCs are more matured under steady state conditions and release of IL-1Ra by epithelial cells suppresses the activation of moDCs (116, 117).

Special attention should be dedicated to the delicate role that IL-1 signaling plays during the cross-talk between keratinocytes and Langerhans cells (LCs) in the skin (**Figure 4B**), which is of fundamental importance during the defense against skin pathogens (118). For example, during development of the neonatal skin, IL-1 signaling serves as a host safeguard mechanism that prevents tolerance to *Staphylococcus aureus (* 119). LCs reside in the epidermis, the outmost layer of the skin, where they are closely associated with keratinocytes. LCs are ontogenically different from dermal DCs and probably arise from a *Mafb*-expressing macrophage progenitor, whereas dermal DCs are more related to the different cDC subsets found in lymphoid tissues. LCs later express the transcription factor *Zbtb46*, which induces a shift towards the cDC identity (118, 120). Keratinocytes are the epidermis’ major cell type and form an important reservoir for IL-1α, which is constitutively expressed in the cytoplasm of these cells (31, 118). IL-1α released from keratinocytes synergizes with GM-CSF to enhance LC survival and capacity to stimulate naive T cells (121–124). Moreover, release of IL-1α following chemically induced keratinocyte damage induces the expression of co-stimulatory molecules (*e.g.* CD80 and CD86), MHC-I and inhibitory receptors (*e.g.* PD-L1/2) on the surface of moDCs. Furthermore, these IL-1α-conditioned moDCs release more pro-inflammatory cytokines (*e.g.* IL-6 and IL-12) and chemokines (*e.g.* IL-8, CCL2 and CXCL9) and appear to be superior in driving CD4^+^ T cell proliferation compared to untreated DCs (114). Next to a clear and well-established role in LC maturation, IL-1R signaling has a known involvement in LC migration to skin-draining LNs, for which the synergy with TNF is essential (125, 126). Indeed, within 2 – 4h after injection of recombinant IL-1β in both murine and human skin, LCs migrate and accumulate in skin-draining LNs (127, 128). The synergy between TNF and IL-1β has been elucidated by experiments that show inhibition of IL-1β-induced LC migration following administration of an anti-TNF antibody and *vice versa (* 129). The current working model suggests that upon receiving a sensitizing trigger, LCs produce IL-1β and as such stimulate keratinocytes to release TNF. In turn, TNF signaling *via* TNFRII provides a first license for LC migration, while a necessary secondary stimulus is delivered *via* autocrine IL-1β signaling (126, 130, 131). Moreover, treatment of mice with an anti-IL-1R1 neutralizing antibody completely abrogates LC migration to skin-draining LNs, as such indicating the importance of this trigger for the information transfer from the innate to the adaptive immune system (132).

Beyond a role in driving maturation of DCs prior to T cell priming, IL-1 signaling additionally mediates DC activation further downstream, for instance by facilitating the CD40/CD40L-interaction during DC/T cell cross-talk. CD4^+^ T cells upregulate CD40L during their activation which is used to interact with CD40 on the DC surface, as such equipping the APC for successful CD8^+^ T cell priming (133). Nakae et al. reported that DC-released IL-1β acts on T cells and upregulates their CD40L and OX40 surface expression (134). In turn, different reports demonstrated that IL-1β facilitates DC activation following the CD40/CD40L-interaction, ultimately leading to DCs that release more pro-inflammatory cytokines (*e.g.* IL-12, IL-1α/β and IL-6) and generate stronger T_H_1 CD4^+^ and CD8^+^ T cell responses (*e.g.* IFN-γ release) (111, 135, 136). However, the ability of T cells to induce release of IL-1β by DCs could be a double-edged sword for the host. For example, during autoimmune myocarditis, IL-1R signaling in DCs promotes the expansion of autoreactive CD4^+^ T cells (137). More recent work demonstrated that during self-reactivity, effector CD4^+^ T cells instruct DCs to release IL-1β *via* an inflammasome-independent mechanism potentiated by TNF and FasL signaling. This TNFR/Fas/caspase 8-dependent pathway is responsible for the systemic inflammation and pathology commonly associated with T cell-driven autoimmune diseases, such as experimental autoimmune encephalitis (EAE) (138). How IL-1 signaling acts during the interplay between DCs and T cells after their initial stimulation, as such further shaping adaptive immune responses, is intriguing and a recent study showed that CD8^+^ T cells also participate in this cross-talk. After antigen presentation, CD8^+^ T cells activate the NLRP3 inflammasome in DCs *via* a perforin-dependent mechanism, which subsequently led to release of IL-1β and further amplification of adaptive immune responses (139). Comparably, maturation and release of fully biologically active IL-1β from bystander cells can be induced by macrophage-derived granzyme A upon recognition of *Pasteurella multocida* toxin (140).

Finally, IL-1 signaling on bystander DC is proposed to potentiate T cell immunity and mediate protective responses during infection with pathogens. In case that pathogen-infected DCs are unable to mount effective adaptive immune responses, successful T cell priming during encounter of live pathogens depends on activation of bystander DCs (141). DCs infected with *Legionella pneumophila* were shown to release IL-1 and IL-1R signaling on bystander cells is essential to overcome infection (142, 143). Data from Akiko Iwasaki’s lab shows that a normal IAV-specific CD8^+^ T cell response can be formed in TLR7^−/−^ and MAVS^−/−^ mice, but fails to develop in IL-1R1^−/−^ mice, which suggests that activation of CD8^+^ T cells upon IAV infection is dependent on the inflammasome-IL-1R axis rather than TLR7 and RIG-I. The authors propose a model where a first DC responds to virus-induced damage to the host by NLRP3 and caspase 1-mediated maturation of IL-1β, which acts on a second bystander DC and promotes its activation, ability to migrate to the draining LN and prime the CD8^+^ T cell response. In this context, DAMP detection *via* the inflammasome/IL-1R axis can thus be perceived as a surrogate for the direct recognition of PAMPs (115). Moreover, IL-1β activity on *Leishmania amazonensis*-infected DCs, which are maintained in a suppressed state, enhances their activation and maturation, which leads to augmented parasite-specific CD4^+^ T cell responses (113).

Together, these combined works provide evidence that IL-1 signaling in DC subsets contributes to their initial maturation, migration and accumulation in lymphoid organs, but also for secondary activation during DC/T cell-crosstalk. During pathogen infection, signaling *via* the IL-1R complex can serve as a surrogate for PAMP recognition, which enables effector T cell responses by activating bystander cells. Next, we will discuss how direct signaling of IL-1 on T cells flavors their differentiation and activation status.

### CD4^+^ T Cells

#### General Aspects

The ability of IL-1 to drive CD4^+^ T cell responses has been reported for decades (111, 135, 144–146). Moreover, defective adaptive immune responses following IAV infection were observed in mice deficient for ASC, caspase 1 and IL-1R1, which all failed to raise effector IAV-specific CD4^+^ T cells and produce neutralizing antibodies (147–149). As addressed above, a first important mode of T cell activation by IL-1 is indirect and depends on the cytokine’s activity on DCs. However, more recent work suggests an important contribution of direct action of IL-1 on CD4^+^ T cells to advance cellular immune responses. Ben-Sasson et al. demonstrated that IL-1α and IL-1β show the same potency to stimulate CD4^+^ T cell responses, both in adoptive T cell transfer (ATCT) as well as in endogenous models. These responses included enhanced CD4^+^ T cell expansion through proliferation, peripheral survival, establishment of memory and memory recall. Moreover, different subsets of CD4^+^ T cells, including T_H_1, T_H_2 and T_H_17, were found to be sensitive for this direct IL-1 activity (150). The innate immune system locally releases inflammatory cytokines as an extra cue beyond TCR triggering and co-stimulation to fine-tune the recall of antigen-experienced effector and memory T cells. A recent study by Jain et al. looked further into how IL-1 drives the reactivation of these subsets and found that IL-1 signaling in memory CD4^+^ T cells of different lineages can license cytokine production. Intriguingly, IL-1R1^−/−^ CD4^+^ T cells differentiated normally into T_H_1, T_H_2 and T_H_17 phenotypes, but failed to produce lineage-specific effector cytokines (*i.e.* IFN-γ, IL-4/5/13, and IL-17A/F/22, respectively) following reactivation. Moreover, this study demonstrated that DCs are a major source of IL-1β, which was found to act directly on CD4^+^ T cells to stabilize different cytokine-encoding transcripts, probably *via* a mechanism dependent on p38 MAPK signaling (7).

Altogether, this indicates that while IL-1 activity clearly facilitates differentiation of naive CD4^+^ T cells following the first antigen encounter, the absolute necessity of IL-1 signaling presumably lies in activation of antigen-experienced effector and memory helper T cells of different lineages. Both TCR triggering and the ligation of co-stimulatory molecules remain the two indispensable instructions that allow for reactivation of these CD4^+^ T cells, but IL-1 signaling can be perceived as an extra signal for further regulation and fine-tuning of these responses. We will summarize how IL-1 signaling modulates differentiation and further activation of the different CD4^+^ T cell subsets.

#### T_H_1 and T_H_2 Immunity

IL-12 is the main cytokine that drives the differentiation of naive CD4^+^ T cells towards an early T_H_1 phenotype after TCR triggering and co-stimulation by inducing the expression of the lineage transcription factor T-bet. While the IL-1 cytokine superfamily member IL-18 cannot induce T_H_1 development itself, its presence in the environment further drives commitment to the T_H_1 lineage, mainly by promoting peripheral T_H_1 proliferation and secretion of IFN-γ, TNF and IL-2. T_H_1 cells also show a remarkably high expression of the IL-18Rα (IL-1R5) (**Table 1**). Generally, T_H_1 cells are well-known for their critical role as initiators of cellular immune responses and as mediators of protection against different intracellular pathogens and generation of anticancer immunity (151). Naive T cells can acquire an early T_H_2 phenotype when the priming environment contains IL-4, which drives expression of the lineage factor GATA3. GATA3 upregulates the expression of the IL-33 receptor ST2 (IL-1R4) on the surface of T_H_2 cells. IL-33 is another IL-1 superfamily member that allows for T_H_2 lineage commitment, proliferation and release of IL-5 and IL-13 (**Table 1**) (18, 151). T_H_2 cells play pivotal roles during infections with helminths and facilitate humoral immune responses, yet, this is now believed to be largely a function of follicular helper T (Tfh) cells (151). Both the study by Ben-Sasson et al. as well as the report by Jain et al. have demonstrated that IL-1 can act directly on both T_H_1 and T_H_2 cells and boost their activation, expansion and effector cytokine production (7, 150). Interestingly, Jain et al. found elevated IL-1R1 expression on antigen-experienced CD4^+^ T cells of different lineages versus their naive counterparts, making the case for a role of IL-1 signaling as inducer of cellular immune responses beyond CD4^+^ T cell priming (7).

IL-1-mediated modulation of the T_H_1/T_H_2 balance has been widely studied in the murine model of cutaneous *Leishmaniasis*. It is widely accepted that during infection with intracellular protozoan parasites from the *Leishmania* genus, T_H_1 responses are associated with resolution of cutaneous lesions and clearance of the pathogen, while T_H_2 responses rather contribute to disease progression (152). Intracellular replication of *L. amazonensis* in macrophages triggers the assembly and activation of the NLRP3 inflammasome, which leads to release of IL-1β and subsequent induction of NO production, a critical mechanism of defense against *Leishmania* species (153). Moreover, mice with deficiencies for several inflammasome components (*e.g.* NLRP3, ASC and caspase 1) fail to control multiplication of *L. amazonensis*, whereas T_H_1 responses against *L. major* are reduced in IL-1R1^−/−^ mice, next to enhanced T_H_2 immunity (153, 154). In accordance with these observations, treatment of BALB/c mice with IL-1α or IL-1β following *L. major* infection induces dramatic reductions in lesion sizes or parasite load due to augmented T_H_1 responses. This IL-1 effect depends on the presence of IL-12 and significantly reduces T_H_2 immunity (155, 156). In addition, infection of C57BL/6 mice with *L. major* leads to enhanced release of the T_H_1-inducing cytokines IL-12, IL-1β and TNF in the infected skin (157).

Different studies employing *Leishmania* infection models unambiguously prove that IL-1 activity promotes T_H_1 responses, while it impairs T_H_2 immunity. However, the exact contribution of IL-1 signaling to the regulation of T_H_2 responses clearly depends on the experimental model system. Infection with the intestinal helminth *Heligmosomoides polygyrus bakeri* promotes the local release of IL-1β, leading to diminished production of IL-25 and IL-33, suboptimal T_H_2 responses and chronic infection (158). Conversely, blocking IL-1 signaling by treatment with the recombinant IL-1Ra in a mouse model of systemic sclerosis enhances T_H_2-mediated inflammation and worsens pulmonary fibrosis (159). Following infection with *Cryptococcus neoformans*, IL-1R1^−/−^ mice show impaired T_H_1 and T_H_17 responses, next to augmented T_H_2 immunity (160). However, mice with an IL-1R1 deficiency on radioresistant lung epithelial cells fail to raise a T_H_2 immune response and do not develop house dust mite-induced asthma, whereas caspase 8-mediated IL-1 activity promotes T_H_2 immunity and contributes to the development of asthma pathogenesis (161, 162). Ovalbumin (OVA)-induced airway hypersensitivity, T_H_2 cytokine production (*i.e.* IL-4 and IL-5) and IgE and IgG1 levels are all reduced in mice with deficiencies in both IL-1α and IL-1β and IL-1R1-deficient mice display diminished CD4^+^ T cell priming in bronchial LNs and lungs (163, 164). On the other hand, NLRP3 activation and IL-1β release do not appear to be required for the activation of T_H_2 immunity by uric acid during asthma development, but this pathway is suggested to be important for skin T_H_2 responses (165, 166). In conclusion, the ability of IL-1 to influence T_H_2 immune responses is clearly context-dependent, as evidenced from studies in different model systems.

Recent work by Kuhn et al. demonstrated that antitumor CD4^+^ T cell responses, including proliferation, tumor infiltration and intratumoral IFN-γ and TNF production, can be promoted by administration of monosodium urate acid crystals in combination with *Mycobacterium smegmatis* and this response completely depends on IL-1R signaling in the host (167, 168). Tumor-specific effector T_H_1 cells establish a pro-inflammatory tumor microenvironment after tumor infiltration and their ability to mediate anti-cancer immune responses depends on IL-1 signaling, which can be delivered by both IL-1α and IL-1β (169, 170).

#### T_H_17 and Treg Immunity

**Figure 5:**
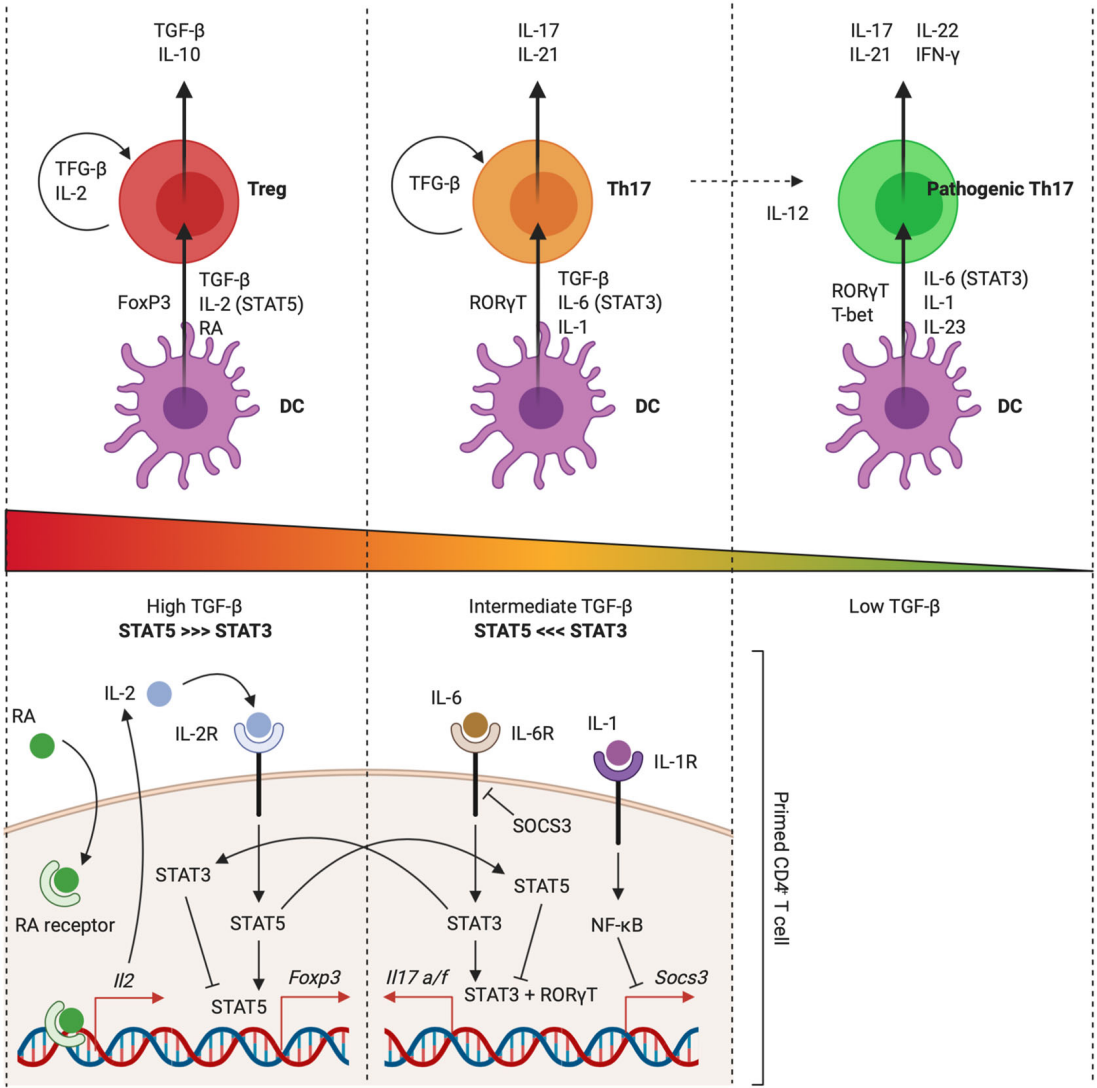
IL-1 signaling facilitates T_H_17 differentiation. TGF-β drives the expression of both FoxP3 and RORγT during CD4^+^ T cell priming. Commitment to a role as regulator or effector is further dependent on IL-2 (STAT5) and IL-6 (STAT3) activity, which antagonize each other. This process was found to be facilitated by other environmental factors, including the metabolite retinoic acid (RA) and the pro-inflammatory cytokine IL-1. Priming environments enriched in TGF-β concentrations and lacking STAT3-dependent cytokines drive Treg development. IL-2 activates STAT5, leading to sustained FoxP3 expression and inhibition of IL-17A and IL-17F production. Presence of RA empowers IL-2 expression and further pushes the balance towards the Treg phenotype. Priming environments that include IL-6 drive STAT3 activation, which mediates IL-17A and IL-17F expression and inhibits FoxP3 expression. IL-1 activates NF-κB, which inhibits expression of SOCS3. This further empowers STAT3 activity and pushes the balance towards the T_H_17 phenotype. In the absence of TGF-β, development of a T_H_1-like T_H_17 phenotype can be driven by IL-6, IL-23 (STAT3), and IL-1. This pathogenic phenotype is characterized by production of IL-17, IFN-γ, IL-21, and IL-22. Figure adapted from (176, 177). Created with BioRender.com.

IL-1 is generally perceived as a cytokine that favors the differentiation of naive CD4^+^ T cells during priming, with a notorious role as a driver of murine and human T_H_17 development. T_H_17 cells are a subset of CD4^+^ T cells that exert potent pro-inflammatory activities, which need to be kept in balance by the dampening anti-inflammatory roles of regulatory T cells (Treg cells). T_H_17 cells express CCR6 and CD103 and preferentially home to mucosal tissues, including the gut (171). T_H_17 cells are essential mediators of mucosal immunity, as they offer local protection against extracellular pathogens, such as opportunistic infections with fungi, and mediate barrier integrity *via* production of IL-17 cytokines. Next to this, T_H_17 cells are strongly implicated during autoimmune responses against self-antigens (172). Intriguingly, T_H_17 and inducible Treg cells are believed to arise from a common progenitor CD4^+^ T cell (172). Upon priming, expression of the transcription factor FoxP3 induces the differentiation of naive CD4^+^ T cells into Treg cells, whereas expression of the transcription factor RORγT enables the generation of T_H_17 cells (173–175). In mice, transforming growth factor (TGF)-β drives the differentiation of CD4^+^ T cells away from T_H_1 and T_H_2 phenotypes by inducing the expression of both RORγT and FoxP3, meaning that a second tier of regulation is necessary to further select between the T_H_17 or the Treg transcriptional programs. Key for this differentiation are cytokines that signal *via* the JAK-STAT pathway: STAT3-inducing cytokines (*e.g.* IL-6, IL-21 and IL-23) drive differentiation towards the T_H_17 phenotype and cytokines that utilize STAT5 (*e.g.* IL-2) promote development of Treg cells (**Table 1** and **Figure 5**) (178).

The minimal requirement for naive murine CD4^+^ T cells to acquire the T_H_17 phenotype is the presence of IL-6 and TGF-β in the priming environment. Although it is generally not regarded as one of the typical polarizing cytokines for mouse T_H_17 differentiation, there is ample evidence in favor of a fundamental role for IL-1 as a facilitator of T_H_17 development in mice (179–181). Basu et al. demonstrated that IL-1 tips the balance between the T_H_17 and Treg transcriptional programs in favor of T_H_17 development by repressing the expression of SOCS3, which is an inhibitor of STAT3 phosphorylation, *via* an NF-κB-dependent mechanism. As such, IL-1 enhances the amplitude and duration of STAT3 phosphorylation downstream of T_H_17-polarizing cytokine signaling, without interfering with STAT5 phosphorylation (182). This leads to enrichment of STAT3 versus STAT5 at their shared consensus sequences, for example in the *Foxp3* locus, which regulates FoxP3 expression, and the *Il17a* and *Il17f* loci, which regulate T_H_17 effector cytokine production (**Figure 5**) (178, 182, 183).

In both mice and human, T_H_17 cells are among the most sensitive immune cells for IL-1 activity due to their relatively high expression of IL-1R1 (10, 11). Abrogated T_H_17 responses and lower incidences of EAE are reported in mice deficient for different players involved in the biology of IL-1, including IL-1R1, IRAK-4, ASC and caspase 1 (184–190). Conversely, mice deficient for IL-1R8 (or SIGIRR), a negative regulator of signaling *via* the IL-1R complex, are more susceptible to EAE due to hyperactivation of T_H_17 cells following immunization with myelin oligodendrocyte glycoprotein peptide (191). Also in experimental arthritis models, IL-1 signaling drives the development of pathogenic T_H_17 cells (192, 193). Uncontrolled and excessive activity of IL-1 in IL-1Ra^−/−^IL-6^−/−^ mice inhibits TGF-β-mediated expression of FoxP3 and induces a T_H_17 transcriptional program in CD4^+^ T cells, which even bypasses the need for IL-6 signaling (194). Interestingly, T cell-specific deletion of IL-1R1 in mouse does not impair T_H_17 development under steady state conditions, but strongly abrogates the potential of T_H_17 cells to migrate and proliferate in the anti-CD3 treatment model (195). Different studies have reported on the capacity of IL-1 to promote differentiation of T_H_17 cells and enhance their expansion, IL-17 production, peripheral survival and capacity to mediate vaccine-induced protection against pathogens, such as *Coccidioides* species and *Blastomyces dermatitidis (* 196–200). Proliferation and persistence of T_H_17 cells following IL-1 signaling is suggested to be a consequence of Akt-mediated activation of the mTOR pathway, which has been linked with cell cycle progression. As a negative feedback switch, T_H_17 cells upregulate IL-1R8 after differentiation, which directly inhibits different signaling pathways induced by IL-1, including phosphorylation of mTOR and JNK (191, 196, 198, 201).

Human T_H_17 differentiation is substantially differently regulated compared to the murine situation, as IL-1 has been demonstrated to be a main polarizing cytokine for T_H_17 development by directly inducing the expression of the lineage transcription factor RORγT in human CD4^+^ T cells. In these studies, differentiation of naive human CD4^+^ T cells towards the T_H_17 phenotype is additionally facilitated by IL-23 or IL-6, but surprisingly antagonized by TGF-β (202, 203). The truth possibly lies somewhere in between and the local concentrations of both TGF-β as well as inflammatory mediators are proposed to be critical determinants of the outcome of T_H_17 development. High local levels of TGF-β can drive the differentiation of naive CD4^+^ T cells towards Treg phenotypes. T_H_17 development is favored in environments that contain pro-inflammatory cytokines, such as IL-6 and IL-1, and are less enriched in TGF-β. Absence of environmental TGF-β or local presence of IL-12 allows the formation of T_H_17 cells with pathogenic phenotypes that typically produce IFN-γ and express T-bet and RORγT (176, 204, 205). A recent study by Grandclaudon et al. demonstrated that IL-1β can synergize with the activity of IL-12 to drive differentiation of naive human CD4^+^ T cells following polyclonal stimulation with CD3/CD28 towards a mixed T_H_1/T_H_17 phenotype, which corresponded with enhanced IL-17F production and expression of RORc, T-bet and IL-23R (206). Both self- and commensal antigens are known as weak triggers of T cell and costimulatory receptors and low-strength TCR and CD28 signals have been reported to favor the differentiation of T_H_17 cells in the presence of cytokines. Revu et al. found that IL-1β synergizes with IL-23 to drive the development of human T_H_17 cells in the absence of CD28 by empowering glucose uptake and glycolytic activity, as such supporting T_H_17 expansion and avoiding anergy (207). Signaling of IL-1β on human Treg cells interferes with alternative splicing of the *Foxp3* transcript by promoting excision of exon 7, which facilitates T_H_17 differentiation *in vitro (* 208). In mice, IL-1 activity was found to attenuate the function of CD4^+^CD25^+^FoxP3^+^ Treg cells, allowing for autoreactive T cells to break tolerance and cause autoimmune disease (209). Administration of increasing amounts of exogenous IL-1β in mice blocks thymic development of Treg cells, which results in decreased amounts of CD4^+^CD25^+^FoxP3^+^ cells and enhanced levels of Treg precursor cells (13). Moreover, T cell-specific ablation of MyD88 abrogated T_H_1 and T_H_17 responses and memory CD4^+^ T cell formation, while no effect could be observed in the Treg subset (210). Besides acting directly on T cells, IL-1β also indirectly drives a T_H_17 instructional program in human immature DCs that leads to upregulation of CD14 and enhancement of a T_H_17-like phenotype in human CD4^+^ memory T cells (211). Human DCs are a major source of IL-1β and addition of NO to LPS-matured human DCs limits their IL-12 production, while enhancing the release of IL-1β, IL-6 and IL-23, thus favoring T_H_17 development (212).

#### Tfh and Tfr Immunity

Tfh cells aid during the differentiation of B lymphocytes into antibody-secreting plasma cells, a process that is negatively regulated by regulatory Tfh (Tfr) cells, which were demonstrated to descend from a Treg lineage (16, 213–216). This cellular interplay is organized in the germinal centers (GCs) of LNs, where Tfh and B cells are located in the middle and Tfr cells build up the surroundings (217). It has been demonstrated that IL-1β acts directly on Tfh cells to enhance their proliferation and induces the production of IL-4 and IL-21, as such initiating a B cell response. Recent work showed that this pathway is strongly maintained by Tfr cells, which express the IL-1 decoy receptor subunit IL-1R2 and actively release IL-1Ra (12). As such, Tfr cells employ a dual homeostatic regulation mechanism to keep IL-1β-mediated activation of Tfh cells in check (12, 16). Barbet et al. identified MHC-II^+^CD11b^+^CD11c^+^CX3CR1^+^ monocytes as the main source of IL-1β upon live vaccine administration. These cells express CCR7, localize to T cell zones in LNs and depend on IFN-β signaling to release IL-1β, which acts directly on Tfh cells and drives their differentiation in response to live bacteria (218). Reduced Tfh responses were also found after sensitization of IL-1R1^−/−^ mice with peanut allergen (219). Also in human systems, the presence of IL-1β in the priming environment enhances Tfh cell development and these responses are diminished upon neutralization of IL-1 activity (220, 221).

Corroborating these recent findings, reduced antibody production following prime/boost immunization has been reported in BALB/c mice with combined deficiencies in IL-1α and IL-1β, while humoral immune responses were enhanced in IL-1Ra^−/−^ counterparts. This work suggests that IL-1 produced by APCs upregulates OX40 and CD40L expression on T cells during priming, as such enhancing their capacity to activate B cells. Despite this, IL-1 appears not be involved in the humoral immune response to T cell-independent antigens (134, 222). In other studies, intact humoral immune responses were observed in C57BL/6 IL-1R1^−/−^ mice following immunization with both T cell-dependent and -independent antigens (154, 223). The contribution of IL-1 activity in humoral immunity is thus controversial. Besides this, the influence of the mouse background, type of antigen used and its delivery method should not be underestimated.

The amount of tumor-infiltrating Tfh cells and B lymphocytes inversely correlates with the progression and recurrence of human colon carcinoma (224). In a recent paper, Roberti et al. demonstrated that in colon carcinoma models, the effect of chemotherapy with oxaliplatin (OXA) on intestinal epithelial cell (IEC) death is dictated by the gut microbiota, which determines the clinical response to anti-PD-1 immune checkpoint blockade. OXA-induced immunogenic IEC death is favored in environments dominated by *Erysipelotrichaceae* family members or *Bacillus fragilis*, which allow for superior responses to anti-PD-1 treatment. This effect relies on migration of CD103^+^CD11b^-^Batf3^+^ cross-presenting DCs to mesenteric LNs, where a Tfh response is primed, which depends on T cell-intrinsic IL-1 and IL-12 signaling. IL-1β-induced Tfh cells promote B cell maturation and class switch and stimulate effector CD8^+^ T cell responses against neo-antigens in the tumor microenvironment (225).

#### T_H_9 Immunity

Approximately a decade ago, IL-9-producing CD4^+^ T cells were first identified and termed T_H_9 cells, which are formed following priming of naive CD4^+^ T cells in the presence of IL-4 and TGF-β (**Table 1**) (226, 227). Over the years, T_H_9 cells have been associated with protection against helminth parasites and were demonstrated to exert very potent antitumor immunity (228–230). In this latter context, a recent report has demonstrated that IL-4 and IL-1β act synergistically in the absence of TGF-β to empower a tumor-specific T_H_9 response, which mediates robust antitumor activity. Of note, the authors showed that direct IL-1 signaling on the CD4^+^ T cell is required for T_H_9 development and they identified the NF-κB pathway as a major signaling cascade that allows for pro-inflammatory IL-9 production (231). Furthermore, ATCT of T_H_9 cells in advanced B16-OVA melanoma tumor-bearing mice revealed that these cells exert potent effector functions, which are distinct from T_H_1 and T_H_17 CD4^+^ T cell subsets, and express less exhaustion markers (*i.e.* PD-1 and LAG3). The proliferative potential of T_H_9 cells appears to be NF-κB-driven, while TRAF6 and Eomes were identified as the main instructors of T_H_9 antitumor effector functions (230).

### CD8^+^ T Cells

The importance of IL-1 signaling for CD8^+^ T cell activation becomes evident from the evolutionary pressure imposed on this pathway by viruses including different vaccinia strains, which encode a viral soluble protein that binds and incapacitates IL-1β. Remarkably, this binding appears to be specific for IL-1β, as IL-1α was found not to be subjected to this neutralization (232–234). Using a modified vaccinia virus Ankara (MVA) that lacks the gene encoding for the anti-IL-1β soluble protein, the lab of Gerd Sutter found enhanced virus-specific memory CD8^+^ T cell responses following prime/boost immunization, which correlated with improved long-lasting protection upon respiratory challenge infection (235, 236). Moreover, MVA-induced murine DCs were identified as the main source for IL-1β production and cytokine secretion following MVA infection is abrogated in DCs with a caspase 1 deficiency, indicating the importance of the inflammasome-IL-1R axis for potent induction of protective virus-specific CD8^+^ T cell immunity (235). In addition, the inflammasome-IL-1R axis appears to be important for successful priming of IAV-specific CD8^+^ T cells, as different reports have demonstrated defective CD8^+^ T cell responses in IL-1R1^−/−^ mice following IAV infection (115, 147, 148). Activation of the NLRP3 inflammasome and subsequent release of IL-1β is also critical during control of infection with West Nile Virus (WNV) (237–239). CD11b^+^CD45^high^ macrophages were identified as the predominant producers of IL-1β in the WNV-infected central nervous system (CNS). While Ramos et al. found less WNV-specific effector CD8^+^ T cells (*i.e.* producing TNF, IFN-γ, perforin and granzyme) in infected IL-1R1^−/−^ mice, Durrant et al. could only link impaired effector CD4^+^ T cells responses to IL-1R1 deficiency during infection (237–239). A follow-up paper by Durrant et al. identified IL-1β as an essential regulator of CXCL12 expression and CXCR4-mediated retention of WNV-specific effector CD4^+^ and CD8^+^ T cells (*i.e.* expressing CD69, IFN-γ and granzyme B) to the blood-brain barrier, as such facilitating their migration into the parenchyma of the CNS (238).

Besides their pivotal work on the role of IL-1α and IL-1β signaling during the development, maintenance and recall of CD4^+^ T cell responses, Ben-Sasson et al. looked into the effects of IL-1 activity on the CD8^+^ T cell compartment (150, 240). Treatment of mice with IL-1β massively enhances the expansion of adoptively transferred OT-I CD8^+^ T cells in both lymphoid (*i.e.* spleen and LNs) and peripheral (*i.e.* lung and liver) organs in response to OVA antigen administration. Next to improved expansion and tissue localization, Ben-Sasson et al. showed that IL-1β empowers CD8^+^ T cell effector functions (*i.e.* production of IFN-γ/granzyme B and cytolytic activity) and memory recall. In addition, the protective capacity of inefficient experimental vaccines against *L. monocytogenes*, vaccinia virus and the lung epithelial TC1 carcinoma is enhanced upon inclusion of IL-1β in the immunization formula (240). Lee et al. combined adoptive transfer of tumor antigen-specific CD8^+^ T cells with repeated IL-1β administration in a murine melanoma model. IL-1β improves the antitumor effect of ATCT, which correlates with stronger infiltration of CD8^+^ T cells in the tumor, a more pronounced effector phenotype and enhanced peripheral survival (241). IL-1R1^−/−^ mice fail to clear infection with lymphocytic choriomeningitis virus (LCMV) and LCMV-specific CD8^+^ T cells from IL-1R1-deficient mice do not synthesize granzymes and lack potent cytolytic activities (242). Despite the fact that CD8^+^ T cell-intrinsic MyD88 expression is essential for adequate expansion and accumulation of antiviral CD8^+^ T cells that control LCMV infection, two independent studies showed that this effect does not depend on signaling *via* IL-1R1 (243, 244). At the peak of the antiviral effector CD8^+^ T cell response to LCMV, impaired expansion, cytokine production (*i.e.* IFN-y, TNF and IL-2) and formation of a CD127^+^ memory precursor pool was observed in IL-1R1^−/−^ CD8^+^ T cells with specificity towards different viral epitopes. Next to the importance of IL-1 signaling in programming potent effector CD8^+^ T cell polyfunctionality, defective formation of a functional LCMV-specific memory CD8^+^ T repertoire was found in IL-1R1^−/−^ mice at 8 weeks after pathogen encounter (245).

The roles of the inflammasome-IL-1R axis and the process of pyroptosis, the form of inflammatory cell death initiated following the formation of membrane GSDM pores after inflammasome activation, have also been studied in the context of mounting anticancer CD8^+^ T cell immunity. A first report by Ghiringhelli et al. showed that the capacity of chemotherapy to induce CD8^+^ T cell responses against developing tumors depends on their ability to trigger the NLRP3 inflammasome in DCs and DC-mediated release of IL-1β. Moreover, the therapeutic response of two independent tumor models to treatment with doxorubicin diminishes upon treatment with an anti-IL-1β, but not an anti-IL-1α neutralizing antibody (246). Delivery of an anti-IL-1β neutralizing antibody also reduces the antitumor properties of other anthracycline chemotherapy in established tumor models (247). On the same note, a recent study by Wang et al. demonstrated a novel method to selectively deliver active GSDMA3 protein to murine tumor cells *in vivo*, as such inducing pro-inflammatory pyroptosis. Intriguingly, pyroptosis levels of less than 15% were found to be sufficient to clear a complete 4T1 mammary carcinoma graft and this effect depends on circulating IL-1β, as inhibition of IL-1β activity using a specific neutralizing antibody completely nullifies antitumor immunity (248). However, no contribution of IL-1β activity in GSDME-mediated immune responses against tumors was observed by Zhang et al. (249).

**Figure 6:**
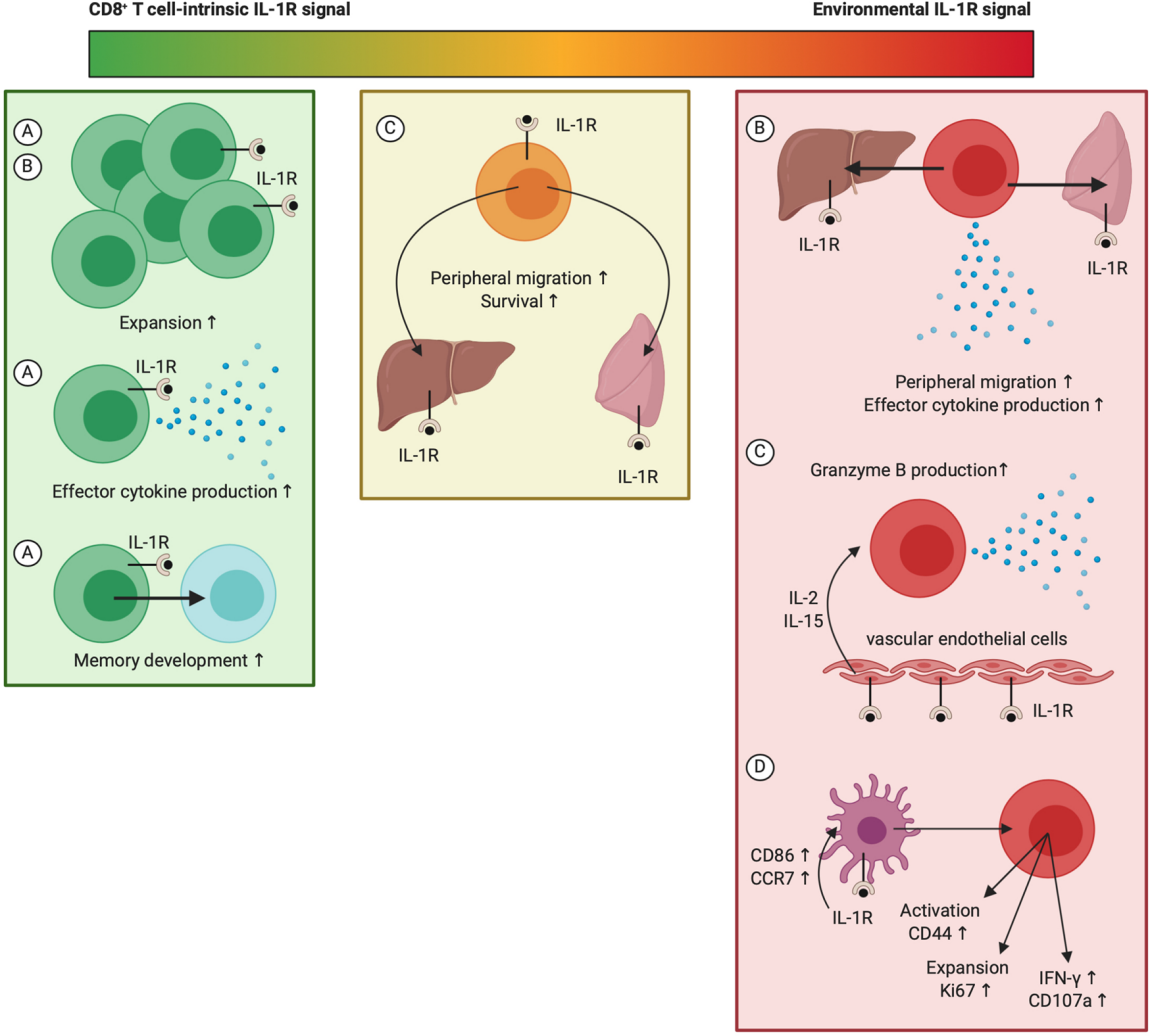
The whereabouts of IL-1R signaling during stimulation of CD8^+^ T cell responses. While IL-1 activity can potently promote CD8^+^ T cell responses at multiple levels, the exact whereabouts of IL-1R triggering for mediation of these effects remain under debate. This figure indicates the context-dependency of several key findings: **(A)** CD8^+^ T cell responses during LCMV-Armstrong infection; **(B)** Treatment of naive mice with antigen and IL-1β following ATCT of CD8^+^ T cells (240); **(C)** Treatment of B16 melanoma tumor-bearing mice that received ATCT of CD8^+^ T cells with IL-1β (241); **(D)** CD8^+^ T cell responses during IAV infection (115). Created with BioRender.com.

While literature provides ample evidence in favor of a stimulatory role of IL-1 for different aspects of CD8^+^ T cell biology, there is ongoing debate on whether IL-1 exerts these actions directly on CD8^+^ T cells or indirectly *via* an intermediate cell type (**Figure 6**). Sarkar et al. reported that intrinsic IL-1 signaling is necessary for the development of effector and memory CD8^+^ T cells responses against LCMV, as both numbers as well as cytokine synthesis were decimated in IL-1R1^−/−^ LCMV-specific CD8^+^ T cells. This indicates that direct IL-1 activity regulates the size and functionality of effector and memory CD8^+^ T cell pools (245). The study by Ben-Sasson et al. shows that IL-1β-mediated expansion of CD8^+^ T cells and their migration into lymphoid organs only requires IL-1R1 expression on OT-I cells, advocating a role for direct IL-1β activity, whereas peripheral localization and cytokine production demands IL-1R1 expression in the host (240). Lee et al. demonstrated that IL-1R1 expression on both the transferred CD8^+^ T cells as well as the host environment is required for the enhancing effect of IL-1β on peripheral accumulation and survival of CD8^+^ T cells. However, IL-1β‘s ability to enhance granzyme B expression in CD8^+^ T cells surprisingly depends on IL-1R1 expression on vascular endothelial cells and host release of IL-2 and IL-15, but not IL-6 and IL-12, nor TCR stimulation. CD11b^+^Ly6G^+^Ly6C^int^ neutrophils in the spleen were additionally suggested as the predominant source of IL-15 (241). While Pang et al. showed that IL-1R1 expression in the hematopoietic cell lineage is an absolute requirement to mount potent anti-IAV CD8^+^ T cell responses, expression of the receptor complex on CD8^+^ T cells is not needed for their activation following infection. In fact, DC-intrinsic IL-1R signaling was found to mediate their upregulation of CCR7 upon IAV infection and their subsequent migration towards draining LNs, where they were shown to prime potent antiviral CD8^+^ T cell immunity (115). Also, in the vaccinia model, reduced CD8^+^ T cell responses were demonstrated in MyD88^−/−^ mice following viral infection, yet, IL-1R1 expression on CD8^+^ T cells does not appear to play a role, according to a study by Zhao et al. (250). From these combined reports, we can at least conclude three important findings considering IL-1R signaling in the regulation of CD8^+^ T cell immunity: (1) both IL-1R1 expression on CD8^+^ T cells as well as peripheral expression of the receptor complex contribute to IL-1-mediated CD8^+^ T cell activation; (2) different bodies of evidence indicate a segregation in this contribution, meaning that the different cellular targets of IL-1 possibly regulate different aspects of the CD8^+^ T cell response; (3) the relative importance of IL-1R signaling in different cell populations could be context-dependent and thus variable in naive mice versus virus-infected or tumor-bearing backgrounds. The recent generation of *Il1r1*-floxed mice is therefore very exciting, as this will allow for detailed dissection of the importance of IL-1R signaling in different cell populations under specific conditions (251).

## Translational Implications

### Vaccines

Prophylactic and therapeutic vaccination strategies are, considering the evidence above, the most obvious clinical applications where the activities of IL-1 could play meaningful roles. Prophylactic vaccination has been used with great success to (nearly) eradicate numerous infectious diseases across the globe, including smallpox, rubella and polio. These strategies depend on evoking potent immunological memory against pathogenic antigens, in such a way that invading pathogens are swiftly eliminated upon encounter (252, 253). The far more experimental therapeutic vaccines have been used primarily in the context of cancer and aim to stimulate T cells that recognize TAAs. TAAs can appear in different forms, which can be highly tumor-specific (*e.g.* neo-epitopes or aberrantly expressed germline antigen genes) or demonstrate rather low tumor specificity (*e.g.* tissue-specific antigens, such as melanocyte proteins, or overexpressed antigens) (254). This can impose an immunological challenge, as certain classes of TAAs are self-antigens for which high-affinity T cells have been eliminated during the immune system’s development, only leaving T cells carrying low-affinity TCRs. Next to this, established tumor masses and immunosuppressive environments are other major hurdles that have been limiting the success of therapeutic cancer vaccination (252).

Vaccines need to display an exquisite safety profile, especially when used prophylactically in healthy subjects. For this reason, traditional vaccines that generally comprise live attenuated or inactivated pathogens have been gradually replaced with recombinant protein subunit formulations, which show an improved safety due to a reduction in their reactogenicity. In order to enhance the efficacy of subunit vaccines, these formulations have been supplemented with adjuvants (*Lat.* “adiuvare” or “to help”). Not only could IL-1 be an interesting candidate for a new vaccine adjuvant as such, but the activities of IL-1α, IL-1β, and other members of the IL-1 cytokine superfamily might be involved in the mode of action (MOA) of several vaccine adjuvants, as extensively summarized in a recent review by Muñoz-Wolf and Lavelle (253). Alum, for instance, which is an overarching denominator for different aluminum-based adjuvants, is known as a potent driver of T_H_2-biased immune responses and antibody production. However, despite being used for nearly a century, alum’s MOA is not completely resolved. Both *in vitro* and *in vivo*, alum has been found to activate the NLRP3 inflammasome and promote the release of IL-1β, but how this exactly influences alum’s adjuvanticity has not been clearly unraveled yet. The relative contribution of IL-1 superfamily cytokines in the alum-mediated immunity could be tissue-dependent and subjected to the susceptibility of resident cells to alum-induced necrosis (253, 255–258). Indeed, Kuroda et al. demonstrated that alum inhalation induces necrosis of CD11c^+^SiglecF^+^ alveolar macrophages and release of IL-1α, which evokes T_H_2-driven airway hypersensitivity. Upon subsequent OVA administration, host IL-1R1 expression was found to be essential in the formation of inducible bronchus-associated lymphoid tissue and antibody production (258). Others have shown that IL-1 superfamily cytokines are unlikely to be prominent regulators of the antibody response induced following alum administration (259–261). Muñoz-Wolf and Lavelle also mention that while different other vaccine adjuvants, including MF59, GLA-SE, AS03 and CAF01, trigger the inflammasome-IL-1 axis, the precise importance of this pathway in the MOA of these adjuvants is incompletely understood (253).

While most of the licensed adjuvant systems mentioned above undeniably raise potent T_H_2 and humoral immune responses, adequate protection against different infectious pathogens (*e.g. M. tuberculosis*, dengue virus or *Plasmodium falciparum*) and cancer requires potent T_H_1, T_H_17 and CD8^+^ T cell immunity (253). In the specific case of IAV, the moderate heterosubtypic immunity that occurs naturally following infection is most likely mediated by cross-reactive T cell responses aimed against highly conserved internal viral epitopes, which makes a case for vaccine adjuvants that promote cellular immunity in the search for a universal IAV vaccine (262, 263). In this context, Lapuente et al. designed an adenoviral vector-encoded IL-1β and co-delivered this intranasally as an adjuvant with vector-encoded IAV nucleoprotein (NP) and hemagglutinin (HA). Next to enhancing HA-specific antibody responses, IL-1β strongly increases both mucosal and systemic NP-specific CD4^+^ and CD8^+^ T cell immunity. Moreover, the CD8^+^ tissue-resident T (T_RM_) cell population induced in the lung was shown to be sufficient for heterosubtypic protection against other IAV strains (264). In a follow-up study, these authors evaluated whether DNA plasmid-encoded IL-1β could act as a cellular adjuvant to improve the efficiency of a DNA vaccine with plasmids encoding the IAV antigens HA and NP. However, while intramuscular co-delivery of IL-1β slightly improved the formation of cross-reactive anti-HA antibodies, no stimulatory effect on NP- and HA-specific CD8^+^ T cells could be observed, suggesting that IL-1β might act as a strictly mucosal adjuvant in IAV vaccination strategies (265).

T_H_17 cells do not only play detrimental roles during autoimmune disease, but also mediate mucosal protection against different pathogens, including extracellular bacteria, *Toxoplasma gondii* and fungi (266–268). Considering the global threat imposed by pathogens such as *M. tuberculosis*, vaccine adjuvants that drive protective mucosal immune responses thus could be of great value (269). The above mentioned CAF01 cationic liposome adjuvant, which is currently under clinical investigation, was found to induce long-lived T_H_17 responses that can be recalled to the lung parenchyma upon challenge infection with *M. tuberculosis* two years after their initial induction (270). Intriguingly, peripheral CAF01-induced T_H_17 cells were found to promote the induction of IgA-producing B lymphocytes in the lung parenchyma and enhanced the formation of GCs and Tfh cells in lung-draining mediastinal LNs (271). Moreover, IL-1R1 signaling appears to be essential for the induction of IFN-γ and IL-17-producing cells in mice following vaccination with CAF01, but not for the generation of an antibody response (272). In an effort to evaluate IL-1β as a possible T_H_17-driving adjuvant, Wüthrich et al. demonstrated that an experimental live attenuated *B. dermatiditis* vaccine supplemented with IL-1β improves T_H_17-mediated protection against subsequent infection (200).

### Cancer Immunotherapy

In the context of cancer, the inflammasome-IL-1 axis can be perceived as a double-edged sword with both pro- and antitumorigenic properties, which is extensively summarized in a recent paper by Rébé and Ghiringhelli (273). There is ample evidence demonstrating that in many different types of cancer, including malignancies in skin, colon and lung, upregulated expression of IL-1β correlates with progression of disease. In addition, several common cancer-associated mutations, such as genetic alterations in *KRAS* and *BRACA1*, promote IL-1β expression. Cancer cells can also drive the production of IL-1β in tumor-associated inflammatory macrophages, as such installing an environment where immune responses are dampened, for instance *via* IL-1β-mediated accumulation of myeloid-derived suppressor cells (MDSCs) that produce IL-10 and recruit pro-tumorigenic neutrophils. Besides this, IL-1β has been described a promotor of angiogenesis and cancer cell metastasis (273). Consequentially, clinical cancer research strongly focuses on antagonizing IL-1 activity in patients, which can be achieved by treatment with NLRP3 inflammasome inhibitors, recombinant IL-1Ra or monoclonal antibodies that neutralize IL-1β (273–275). The CANTOS (for “canakinumab anti-inflammatory thrombosis outcome study”) trial evaluated the effect of treatment with canakinumab, an anti-IL-1β neutralizing monoclonal IgG1 antibody, in patients with a history of myocardial infarction and high circulating levels of C-reactive protein (CRP). The study found that treatment with canakinumab could dose-dependently reduce levels of CRP and the risk of recurrence of a cardiovascular event (276). Moreover, treatment with canakinumab significantly lowered the incidence and mortality of lung cancer, making a case for strategic inhibition of IL-1 activity in patients at risk (277).

In contrast to this, IL-1 activity can promote anti-tumor immune responses by influencing both DCs and T cells in different models and experimental settings. Therapeutic vaccination of mice with irradiated melanoma cells that secrete IL-1β impairs tumor growth, which correlates with enhanced T cell activity (*i.e.* more release of IL-2/IFN-γ and augmented cytolytic activity upon *ex vivo* restimulation) (278). IL-1R triggering activates bystander DCs, which could be of importance for the improvement of DC vaccination strategies (143). Indeed, different studies have shown that successful priming of CD8^+^ T cell responses during vaccination with *ex vivo* matured and antigen-loaded DCs is dependent on help from bystander DCs (279, 280). Allogeneic DCs that are matured *ex vivo* with a cocktail containing poly(I:C), R848 and IFN-γ in combination with an infection-enhanced adenoviral vector produce high amounts of IL-1β. This correlates with an enhanced potential to activate bystander DCs, which in turn cross-present antigen to CD8^+^ T cells and induce an antigen-specific adaptive immune response (281). In the CT26 colon carcinoma model, systemic administration of *Salmonella typhimurium* in mice enhances the release of IL-1β by DCs and inhibits tumor growth, which can be reverted upon treatment with an IL-1β-neutralizing antibody (282, 283). Work by Segovia et al. demonstrated that mice with a deficiency for the transmembrane protein 176B (TMEM176B), which inhibits activation of the NLRP3 inflammasome by controlling the cellular Ca^2+^ flux, efficiently control tumor growth in a caspase 1 and IL-1β-dependent manner. Moreover, pharmacological targeting of TMEM176B mediates inhibition of tumor growth and increases susceptibility to immune checkpoint inhibition (284). Recent work by Zhivaki et al. shows that hyperactive cDC1s produce mature IL-1β in a NLRP3-dependent, but pyroptosis-independent manner and display an enhanced ability to take up and present TAAs, migrate to tumor-draining LNs and promote antitumor effector CD8^+^ T cells, altogether establishing a durable antitumor response. Interestingly, neutralization of IL-1β activity with an antibody completely abrogates the antitumor response mediated by these hyperactive cDC1s (70).

IL-1 could be a strong candidate to improve the therapeutic efficacy of adoptive T cell transfer in cancer, which remains largely ineffective for the majority of patients carrying solid tumors. The general idea is that cancer patients receive a transfer of T cells with reactivity against different tumor antigens, which can be either inherent or acquired following genetic insertion of transgenic TCRs or chimeric antigen receptors (CARs) (285). In this context, Lee et al. showed that repeated administration of IL-1β improves the antitumor activity of ATCT in a mouse model of melanoma, while Sarkar et al. found that IL-1 supplementation enhances priming and expansion of anti-CD19 human CAR-T cells (241, 245).

In conclusion, while IL-1 activity primarily shows pro-tumorigenic properties, different bodies of evidence demonstrated that antitumor immune responses can be facilitated by IL-1. Therapeutic application of IL-1’s activity in the context of cancer will completely depend on the ability to clearly segregate its pro-tumorigenic from its antitumorigenic effects.

### Toward Safe and Efficient Clinical Application of IL-1

Efficient clinical translation of cytokine activity is hampered by a variety of problems. First, long-term and high-dose cytokine administration might raise detrimental side effects. In the case of IL-1α and IL-1β, these toxicities include fevers, rigor and headaches. Hypotension is the dose-limiting factor and the maximum tolerated dose (MTD) of intravenously administered IL-1β was found to range from 0.07 – 0.3 μg/kg body weight (286). A second problem that some cytokines face is the necessity of dose escalation to reach sufficient therapeutic efficacy, which is due their limited serum half-life (287). Third, cytokines are highly pleiotropic molecules and not all of their functionalities are desirable. The abovementioned tumor-promoting functions of IL-1 could therefore nullify the favorable promotion of an antitumor CD8^+^ T cell response (273). Isolation of the favorable immunostimulatory properties of IL-1 from its undesired side effects might pave the way for its safe and efficient clinical application. In this regard, structure/function analysis has revealed a region within IL-1β (*i.e.* the primary sequence VQGEESNDK located between β-strand four and five) that displays comparable adjuvanticity to complete IL-1β in the absence of inflammation and toxicity (288). An alternative solution for the abovementioned problems are so-called immunocytokines, which represent fusion proteins comprised of a WT cytokine and a targeting moiety directed against a cell type-specific surface molecule. Immunocytokines aim to widen the therapeutic window of cytokines by increasing their local concentration at carefully selected target sites. This empowers the local biological activity of the cytokine moiety and allows for the use of lower amounts of immunocytokine to achieve the same therapeutic effect compared with the WT cytokine only, as such reducing cytokine-related toxicities (289). As of today, one IL-1β-based immunocytokine has been preclinically evaluated. This immunocytokine delivers IL-1β activity to the tumor microenvironment and modestly slows down the subcutaneous growth of a murine B16 melanoma tumor (290). A limitation of immunocytokines is the fact that these still comprise WT cytokines that maintain the capacity to exert undesired cytokine activities on non-targeted cells (291, 292). A modification of the immunocytokine concept are AcTakines (for “Activity-on-Target cytokines”), which represent fusion molecules that carry a mutation in the cytokine moiety, as such rendering them essentially inactive. Upon delivery of the mutated cytokine to a cell type-specific surface molecule, cytokine activity can be restored up to WT level. As such, AcTakines are devised to remain inactive *en route* through the body and reveal their full agonist activity following binding to selected cell types only (293). AcTakines based on type I IFN (294, 295) and TNF (296) activity were previously demonstrated to mediate potent antitumor activity without raising the toxicities observed with WT cytokine administration. Recently, we developed an AcTakine molecule that delivers IL-1β activity to CD8^+^ T cells and safely promotes T cell activation, proliferation and memory differentiation. Inclusion of this AcTakine in an experimental prime/boost IAV vaccine established protection against a high influenza challenge dose, which correlated with the formation of a potent and durable IAV-specific cellular immune response (297). The use of this strategy could be expanded to other models, for instance to evaluate antitumor CD8^+^ T cell activation by IL-1β.

## Closing Remarks and Open Questions

The pro-inflammatory cytokines IL-1α and IL-1β can strongly impact the outcome of adaptive immune responses. However, their pleiotropic activities are usually not included in the three-signal model, an empirical framework that places adaptive immunity under innate control. Successful progression from productive to actual protective T cell immunity requires a more holistic view, with additional attention for the biological effects of these pro-inflammatory IL-1 cytokine superfamily members. A revised model could be of major importance for more rational development of adjuvants that promote cellular immune responses, which are highly requested in vaccination against intracellular pathogens and the new generation of recombinant personalized tumor vaccines.

As IL-1α and IL-1β utilize the exact same receptor complex, their biological activities can be regarded as redundant. However, clear temporal and spatial factors distinguish these two pro-inflammatory mediators: whereas IL-1α performs a local role as an alarmin that is released upon cellular damage, the release of IL-1β, which can also appear in circulation, is strictly controlled. From this biological perspective, the activities of cytokine alarmins, including IL-1α and the IL-1 cytokine superfamily member IL-33, could be important to induce in mucosal vaccination strategies. As many pathogens enter the host *via* mucosal membranes of the respiratory, genital and gastro-intestinal tract, raising local immune responses at the sites where infection and transmission take place is highly requested and the success of mucosal vaccination to locally induce functional T cell responses will in part depend on the availability of powerful adjuvants that yield such responses.

The IL-1 cytokine superfamily contains other members besides IL-1α and IL-1β with functionalities that could be used to fine-tune adaptive immune responses. IL-33 is an established inducer of T_H_2 immunity and promotes the release of T_H_2-polarizing cytokines *in vivo (* 298). IL-18 was originally described as “IFN-γ-releasing factor” due to its ability to promote T_H_1 responses and NK cell activation (299). Further research is requested on how these cytokine properties can be exploited to advance immunization strategies.

## Author Contributions

BVDE wrote the manuscript and assembled the figures. JT and SG revised the manuscript. All authors contributed to the article and approved the submitted version.

## Funding

BVDE is a doctoral fellow receiving FWO-SB funding. This work was supported by an FWO grant (project G045318) awarded to JT and SG and a UGent Methusalem and an Advanced ERC grant (CYRE 340941) awarded to JT. The Receptor Research Laboratories headed by JT. received financial research support from Orionis Biosciences BV.

## Conflict of Interest

JT is affiliated with Orionis Biosciences BV as scientific advisor and holds equity interests in Orionis Biosciences BV.

The remaining authors declare that the research was conducted in the absence of any commercial or financial relationships that could be construed as a potential conflict of interest.
